# Self‐Trapped Excitons Activate Pseudo‐Inert Basal Planes of 2D Organic Semiconductors for Improved Photocatalysis

**DOI:** 10.1002/adma.202505653

**Published:** 2025-05-16

**Authors:** Jindi Yang, Xiangkang Zeng, Bicheng Zhu, Sharidya Rahman, Chuanbiao Bie, Ming Yong, Kaige Sun, Mike Tebyetekerwa, Zhuyuan Wang, Lijun Guo, Xin Sun, Yuan Kang, Lars Thomsen, Zhimeng Sun, Zhongguo Zhang, Xiwang Zhang

**Affiliations:** ^1^ Dow Centre for Sustainable Engineering Innovation School of Chemical Engineering The University of Queensland St Lucia Queensland 4072 Australia; ^2^ Laboratory of Solar Fuel Faculty of Materials Science and Chemistry China University of Geosciences 68 Jincheng Street Wuhan 430078 P. R. China; ^3^ ARC Centre of Excellence in Exciton Science Department of Materials Science & Engineering Monash University Clayton VIC 3800 Australia; ^4^ Department of Chemical and Biological Engineering Monash University Clayton VIC 3800 Australia; ^5^ Australian Synchrotron 800 Blackburn Road Clayton VIC 3168 Australia; ^6^ Institute of Resources and Environment Beijing Academy of Science and Technology North Xisanhuan Road 27, Haidian District Beijing 100089 China; ^7^ ARC Centre of Excellence for Green Electrochemical Transformation of Carbon Dioxide The University of Queensland St Lucia Queensland 4072 Australia

**Keywords:** 2D materials, hydrogen peroxide production, organic 2D semiconductor, photophotocatalysis, surface reaction

## Abstract

2D organic semiconductors are widely considered superior photocatalysts due to their large basal planes, which host abundant and tunable reaction sites. However, here, it is discovered that these basal planes can be pseudo‐inert, fundamentally challenging conventional design strategies that assume uniform activity on the surface of 2D organic semiconductors. Using 2D potassium‐poly (heptazine imide) (KPHI) for hydrogen peroxide photocatalysis as a model, it is demonstrated that the pseudo‐inertness of basal planes stems from preferential exciton transport to edges, instead of interlayer transport in highly ordered structures. Thus, their dimension reduction enables controlled localization of exciton due to the self‐trapping mechanism, whereby the basal planes can transform from pseudo‐inert state into active catalytic sites. With this knowledge, a modified 2D KPHI capable of generating 35 mmol g^−1^ h^−1^ of H_2_O_2_, which is over 350% increase compared to pristine KPHI, is reported. More interestingly, the activated basal planes promote H_2_O_2_ production through a reaction pathway distinct from that of pseudo‐inert basal planes. These findings establish fundamental principles connecting crystal structure, exciton dynamics, and reactive site distribution, providing new insights into the design of high‐performance photocatalysts.

## Graphical abstract

2D organic semiconductors are widely believed to have uniformly active surfaces. This study reveals that their basal planes can be pseudo‐inert due to anisotropic exciton migration. By inducing exciton self‐trapping through dimensional reduction, these inert planes are activated, boosting hydrogen peroxide production by over 350%. The work uncovers key structure–property relationships for advanced photocatalyst design.

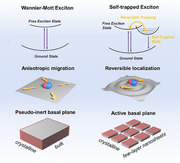

## 1 Introduction

Photocatalysis offers a sustainable approach to harness solar energy for chemical transformations,^[^ ^1^ ^]^ wherein the abundance of reactive sites on catalysts critically determines catalytic efficiency.^[^ ^2^ ^]^ 2D materials have emerged as promising photocatalysts owing to their expansive basal planes, which were initially anticipated to provide abundant reactive sites.^[^ ^3^ ^]^ However, studies of inorganic 2D semiconductors have revealed significant limitations in their basal‐plane activity. In materials such as MoS_2_ and ZnIn_2_S_4_, the basal planes contain fully saturated orbitals, which render these extensive surfaces catalytically inactive.^[^ ^4^ ^]^ These surfaces are often regarded as intrinsically inert, inherently lacking reactive sites, because their unfavorable electronic structures impede reactant adsorption. Strategies including defect engineering and heteroatom doping have been pursued to activate these intrinsically inert basal planes, substantially enhancing catalytic performance.^[^ ^5^ ^]^ These advances highlight the critical importance of understanding and controlling basal‐plane activity.

In contrast, organic 2D photocatalysts—including carbon nitrides^[^ ^6^ ^]^ and covalent organic frameworks^[^ ^7^ ^]^—have long been considered to possess intrinsically active basal planes. This assumption derives from their modular molecular structures and diverse electronic properties, which ostensibly allow for tailored design of reactive sites.^[^ ^8^ ^]^ While these basal planes indeed possess intrinsic reactivity and can adsorb reactants, a crucial aspect has been overlooked: effective photocatalysis requires accessibility of photogenerated charge carriers at these sites.

**Figure 1:**
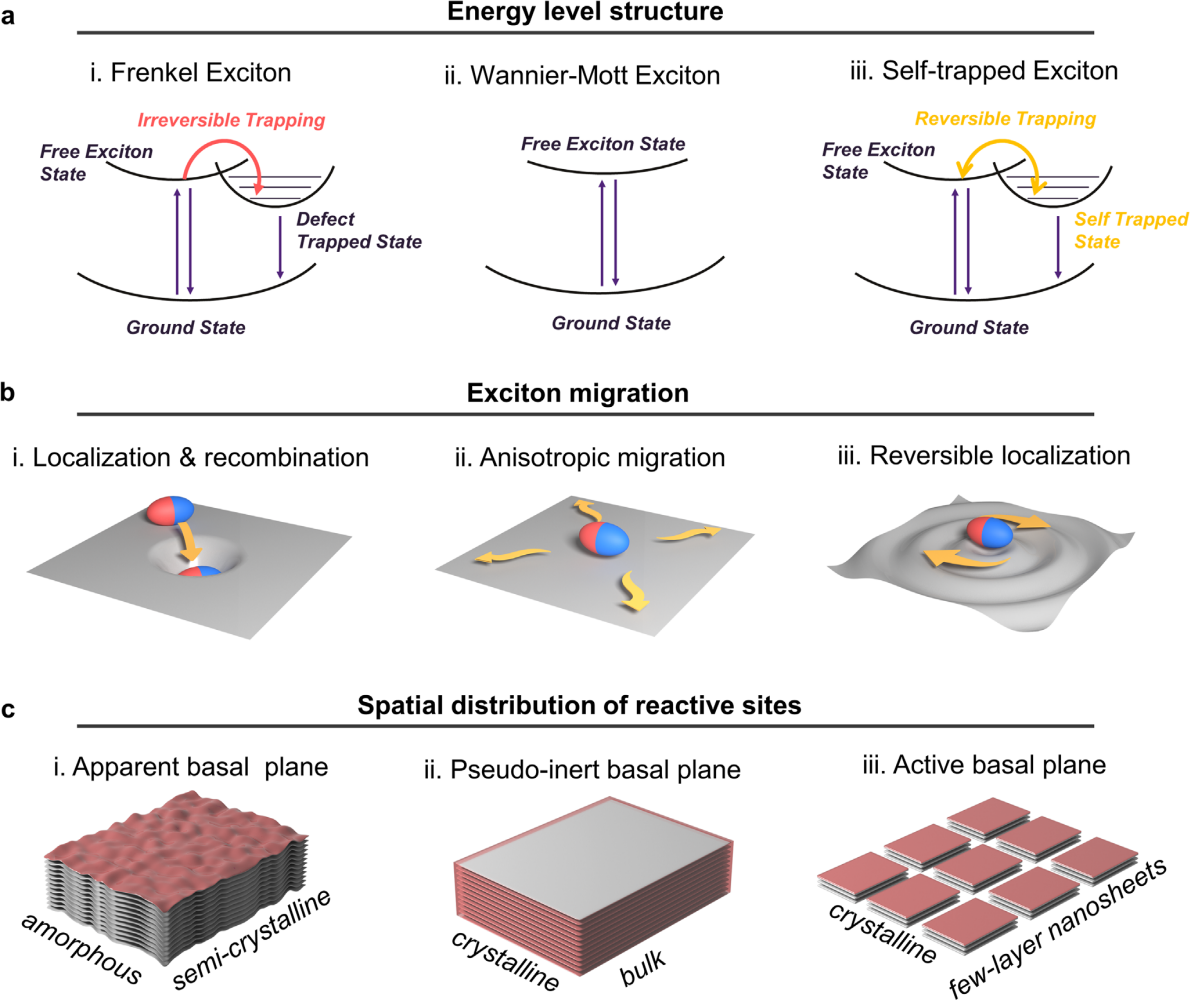
Crystal structure control of exciton transport dictates basal‐plane activity in 2D organic photocatalysts. Schematic diagram of a) energy level structure, b) exciton migration (ellipsoids in red and blue represent tightly bound electron‐hole pairs characteristic of organic semiconductors), and c) spatial distribution of reactive sites in organic 2D semiconductors with different crystal structures (the red regions indicate catalytically active areas). In amorphous/semi‐crystalline structures (panel i), localized Frenkel excitons are trapped by structural defects, leading to bulk recombination. Only surface‐generated excitons participate in reactions, producing apparent basal‐plane activity. In highly crystalline structures (panel ii), ordered lattices enable Wannier‐Mott excitons to delocalize across hundreds of unit cells. The combination of strong in‐plane bonds and weak interlayer interactions drives preferential in‐plane exciton transport to edge sites. This creates pseudo‐inert basal planes: surfaces with abundant catalytic sites that remain inactive due to insufficient photogenerated charge carriers. Dimensional reduction to nanosheets enables beneficial exciton localization through reversible self‐trapping (panel iii). Unlike permanent structural defects, this dynamic process creates temporary trapping sites through exciton‐phonon coupling, allowing controlled exciton localization while avoiding excessive recombination.

We propose that carrier dynamics fundamentally determines basal‐plane activity of 2D organic semiconductors, creating what we define as “pseudo‐inert” surfaces. Unlike intrinsically inert surfaces that lack reaction sites entirely, pseudo‐inert surfaces possess potential reaction sites but remain catalytically inactive due to insufficient charge carrier supply. In organic semiconductors, low dielectric constants cause strong Coulombic binding of photoexcited electron‐hole pairs into excitons.^[^ ^9^ ^]^ These excitons behave distinctly in crystalline versus amorphous structures.^[^ ^8a^ ^]^ In amorphous or semi‐crystalline materials (**Figure** **1**–i), structural defects constrain long‐range transport,^[^ ^10^ ^]^ localizing excitons near their generation sites.^[^ ^11^ ^]^ This localization results in apparent basal‐plane activity, whereas bulk excitons remain unutilized.^[^ ^12^ ^]^ Such apparent basal‐plane activity has guided catalyst design and has been validated by numerous experimental studies.^[^ ^13^ ^]^ Conversely, in highly ordered materials (Figure 1–ii), the anisotropic structure—featuring continuous covalent bonding within planes and weak van der Waals interactions between layers—enables efficient long‐range exciton transport to edge sites rather than across layers.^[^ ^14^ ^]^ This creates pseudo‐inert basal planes, where intrinsically reactive sites remain catalytically inactive due to insufficient charge carrier supply. Indeed, recent studies of crystalline poly (triazine imide) precisely revealed this behavior, with edge planes showing higher photocatalytic activity than their basal planes.^[^ ^15^ ^]^ The crystallinity‐dependent behavior reconciles contradictory results of basal‐plane activity and reveals potentially widespread pseudo‐inertness in crystalline organic 2D photocatalysts. This may fundamentally challenge conventional design strategies that assume uniformly distributed active sites in 2D photocatalysts.

To validate the hypothesis that exciton dynamics governs basal‐plane activity of 2D organic photocatalysts, we designed a comparative system using highly crystalline potassium‐poly(heptazine imide) (KPHI) and amorphous potassium‐polymeric carbon nitride (KPCN).^[^ ^6^, ^16^ ^]^ Photodeposition experiments mapped the spatial distribution of reactive sites, confirming basal‐plane inertness in KPHI and basal‐plane activity in KPCN. More surprisingly, we discovered that exfoliation of bulk‐KPHI to layered nanosheets (nano‐KPHI) activated the pseudo‐inert basal planes. Photophysical tests and ultrafast absorption spectroscopy revealed reversible exciton self‐trapping in nano‐KPHI. This self‐trapping restrains long‐range exciton migration while preventing extensive recombination, as conceptually illustrated in Figure 1–iii. We demonstrated the practical impact of basal‐plane activation through photocatalytic oxygen reduction for H_2_O_2_ production, achieving 35 mmol g^−1^ h^−1^ with nano‐KPHI. Mechanistic studies identified distinct reaction pathways: indirect two‐electron transfer on active basal planes and direct two‐electron transfer on pseudo‐inert basal planes. This work establishes clear links between crystal structure, exciton transport, and basal‐plane reactivity, providing new principles for designing high‐performance photocatalysts.

## 2 Results and Discussion

### 2.1 Materials Synthesis and Characterization

To obtain the desired photocatalysts, we synthesized potassium‐intercalated carbon nitride using an improved ionothermal method^[^ ^16^, ^17^ ^]^ and controlled the crystal structure by achieving thermal polymerization temperature (Figure S3, Supporting Information). Sequentially increasing reaction temperature transformed the product from amorphous KPCN (550 °C) to highly crystalline bulk‐KPHI (575 °C), followed by controlled fragmentation through strategic breaking of imine bonds (600 °C).^[^ ^18^ ^]^ This size reduction weakens interlayer van der Waals forces, enabling spontaneous exfoliation into a few‐layer nanosheet (nano‐KPHI) during aqueous processing. By maintaining the crystalline structure while reducing dimensions, nano‐KPHI was initially expected to enhance edge‐based catalysis through increased edge‐to‐surface ratio and shortened exciton transport distances to edge sites.

Morphological analysis confirmed the successful synthesis of the designed photocatalysts. Scanning electron microscopy (SEM, Figures S4–S6, Supporting Information) provided an overview of the structural evolution from bulk materials to thinner lamellae, while atomic force microscopy (AFM) offered quantitative details on particle dimensions and thickness. AFM analysis specifically showed that nano‐KPHI primarily consists of few‐layer nanosheets with a thickness in the range of 1.5–5 nm and lateral dimensions typically ranging from tens of nanometers (Figure S7, Supporting Information). In contrast, bulk‐KPHI and KPCN exhibited typical bulk characteristics with lateral dimensions from several hundred nanometres up to 1.5 µm (Figures S8 and S9, Supporting Information), and thicknesses ranging from ≈120–500 nm for bulk‐KPHI and 200 nm–1 µm for KPCN. These combined SEM and AFM observations confirm the successful dimensional reduction achieved in nano‐KPHI.

**Figure 2:**
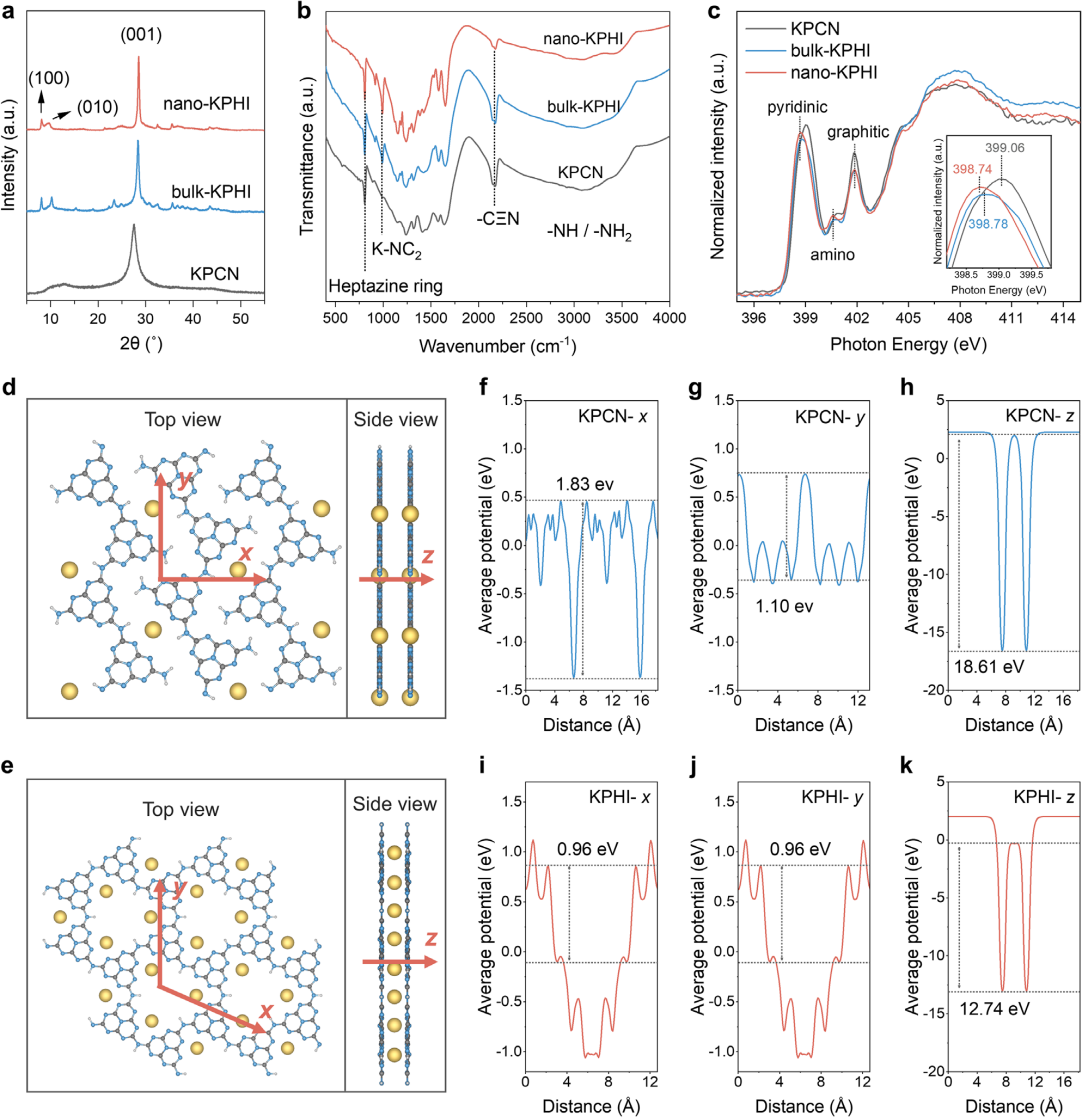
Structural characterization and directional electron transport properties. a) XRD patterns, and b) FTIR spectra of KPCN (gray), bulk‐KPHI (blue) and nano‐KPHI (red). c) N K‐edge XANES of KPCN (gray), bulk‐KPHI (blue) and nano‐KPHI (red). The positions of pyridinic nitrogen and amino/imino nitrogen are marked in the figure, and the inner figure is a local enlargement of the pyridinic nitrogen peak (left) and amino/imino nitrogen peak (right). Schematic diagram of d) KPCN, and e) KPHI structure, top view (left) side view (right). The directions for electrostatic potential calculation are aligned with the periodic directions of the unit cell and are marked with red arrows. The calculated electrostatic potentials for KPCN along f) *x*‐direction, g) *y*‐direction, and h) *z*‐direction, and for KPHI along i) *x*‐direction, j) *y*‐direction, and k) *z*‐direction. The maximum potential differences between adjacent atomic columns are marked on the diagram to evaluate the electron transport capacities along these directions.

Transmission electron microscopy (TEM, Figures S10–S12, Supporting Information) further corroborated these morphological findings and provided high‐resolution evidence of the materials' crystalline structure. High‐resolution TEM imaging demonstrated the preserved crystallinity in both bulk and nano‐KPHI, evidenced by clear lattice fringes with a 1.02 nm spacing characteristic of the (100) crystallographic facet, while KPCN showed typical amorphous features without an ordered structure. As illustrated in **Figure** **2**a, X‐ray diffraction (XRD) analysis revealed broad, diffuse peaks for amorphous KPCN in contrast to the sharp, well‐defined diffraction features of crystalline KPHI. The (002) diffraction peak of KPHI displays a reduced peak width and lower angle position, indicating superior crystalline order and tighter interlayer stacking.^[^ ^19^ ^]^

X‐ray photoelectron spectroscopy (XPS, Figure S13, Supporting Information) confirmed the successful synthesis of our materials, identifying C, N, and K as primary constituents with potassium in ionic form.^[^ ^19a^ ^]^ The consistent C 1s and N 1s peak profiles across samples demonstrated preserved chemical frameworks (Figure S14, Supporting Information), while the negligible Cl signals indicated efficient removal of excess KCl used during synthesis (Figure S15, Supporting Information). Fourier transform infrared spectroscopy (FT‐IR, Figure 2b) revealed that both KPCN and KPHI share the same basic heptazine‐based framework, as evidenced by characteristic features including heptazine ring modes (809 cm^−1^) and conjugated heterocycle features (1200–1700 cm^−1^).^[^ ^16a^ ^]^ The primary structural distinction lies in their K^+^ coordination patterns, as indicated by a distinct K‐NC_2_ symmetric vibration (986 cm^−1^) in KPHI samples.^[^ ^16a^ ^]^

Previous studies have demonstrated that ion coordination patterns play a crucial role in determining exciton transport behavior,^[^ ^14^, ^20^ ^]^ warranting careful examination of these structural features. Synchrotron‐based near‐edge X‐ray absorption fine structure (NEXAFS) spectroscopy provided detailed structural information about K^+^ coordination.^[^ ^21^ ^]^ Figure 2c presents the N K‐edge spectra, showing a significant shift in the pyridinic N peak for KPHI, indicating strong K^+^‐induced charge transfer.^[^ ^18^, ^22^ ^]^ The C K‐edge spectra revealed bifurcation of the N─C═N peak (Figure S16, Supporting Information), demonstrating π‐electron redistribution from cyan amino groups to pyridinic N sites,^[^ ^22^, ^23^ ^]^ which is consistent with the FTIR results of unique K^+^‐pyridinic N coordination in KPHI. Additionally, the evolution from amino to imino groups in N K‐edge spectra and reduced defect‐related signals in C K‐edge spectra confirmed the higher crystalline order and crosslinking degree in KPHI samples.^[^ ^22^ ^]^

### 2.2 Anisotropy of Exciton Transport

Based on our comprehensive characterization results and previous investigations of carbon nitride structures,^[^ ^14^, ^17^, ^18^, ^24^ ^]^ we constructed detailed structural models for computational analysis. For KPCN, we adopted a model featuring incompletely polymerized heptazine chain segments with K^+^ ions intercalated between the chains (Figure 2d; Figure S17, Supporting Information), reflecting its low degree of polymerization and amorphous nature. For both bulk and nano‐KPHI, which share an identical chemical structure, we used a model consisting of a complete 2D covalent network with K^+^ ions coordinated to pyridinic nitrogen between the layers (Figure 2e; Figure S18, Supporting Information).

To understand the characteristics of anisotropic exciton transport, we performed density functional theory (DFT) calculations analyzing intra‐ and interlayer electrostatic potentials. The exciton mobility can be evaluated through potential differences between neighboring atomic columns, where smaller differences indicate more facile electron transport.^[^ ^14a^ ^]^ In KPCN, the potential barrier along the *x* direction (1.83 eV, Figure 2f), characterized by noncovalent hydrogen bonds, significantly exceeds that along the fully covalent *y* direction (1.10 eV, Figure 2g). KPHI, with its structural symmetry, displays uniform electrostatic potential distribution in both *x* and *y* directions (Figure 2i,j), exhibiting a maximum potential barrier of 0.96 eV across nonconjugated amine bonds. Analysis of interlayer transport revealed that both materials show substantially greater potential barriers in the *z*‐direction than their respective intralayer differences do. The interlayer potential difference in KPHI (12.74 eV, Figure 2k) is notably lower than that in KPCN (18.61 eV, Figure 2h), attributed to the different K^+^ intercalation patterns. Charge density calculations further illuminate these differences: KPHI exhibits continuous charge distribution across its carbon and nitrogen framework (Figure S19, Supporting Information), while KPCN shows distinct vacuum gaps between chains(Figure S20, Supporting Information), partially compensated by intralayer K^+^ insertion.^[^ ^15a^ ^]^ The interlayer region of KPCN presents a typical van der Waals gap, whereas KPHI develops a more continuous cross‐layer charge distribution due to K^+^ intercalation between layers (Figure S21, Supporting Information).^[^ ^20a^ ^]^

These calculations reveal that both materials exhibit inherent anisotropy, favoring in‐plane electron movement over interlayer transport. Notably, in KPCN, the gaps between incompletely polymerized segments may impede in‐plane exciton transport, with amino groups potentially serving as recombination sites. This observation aligns with previous predictions about the impact of structural discontinuities on exciton transport in carbon nitride materials.^[^ ^25^ ^]^ From these analyses, we predict that KPCN will exhibit basal‐plane activity due to localized excitons at structural discontinuities, while KPHI's highly ordered structure and extended π‐electron system will likely result in pseudo‐inert basal planes as excitons efficiently migrate to edge sites.

### 2.3 Spatial Distribution of Reactive Sites

**Figure 3:**
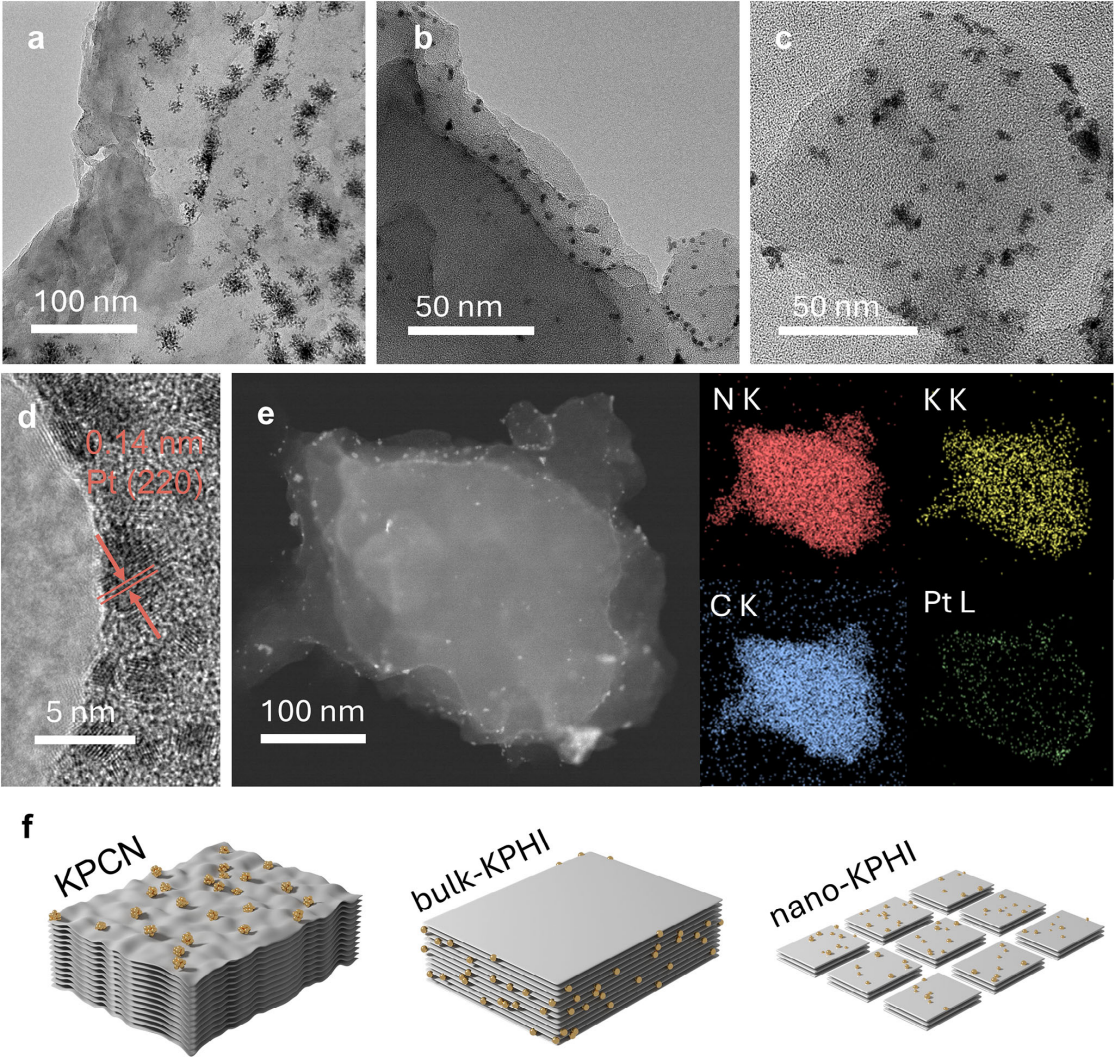
Photodeposition method for determining the spatial distribution of reactive centres. TEM images of a) KPCN, b) bulk‐KPHI, and c) nano‐KPHI after photodeposition. d) HR‐TEM images of bulk‐KPHI with Pt nanoparticles deposited at the edges. e) The HAADF‐STEM image and EDS maps of bulk‐KPHI with Pt deposition. f) Schematic illustration of Pt particles distribution in KPCN, bulk‐KPHI, and nano‐KPHI.

Photo‐deposition experiments provided direct validation of our theoretical predictions regarding reactive site distribution. Amorphous KPCN exhibited significant basal‐plane activity (**Figure** **3**a), which is consistent with previous observations.^[^ ^13^, ^26^ ^]^ The clustered distribution of Pt nanoparticles on KPCN reflects its constrained exciton transport, where localized electric fields at initial deposition sites trap subsequent excitons (Figure S22, Supporting Information). In contrast, bulk‐KPHI displayed clear evidence of basal‐plane inertness (Figure 3b; Figure S23, Supporting Information), with Pt nanoparticles preferentially depositing along edges of the nanosheets (Figure 3d). High‐angle annular dark‐field scanning transmission electron microscopy (HAADF‐STEM) imaging of bulk‐KPHI after photodeposition revealed bright Pt nanoparticles systematically concentrated at the edges (Figure 3e), strongly supporting our computational prediction of anisotropic exciton transport directing carriers toward edge sites.

Surprisingly, nano‐KPHI, despite sharing the same chemical framework as bulk‐KPHI, demonstrated uniform Pt nanoparticle distribution across its basal surface (Figure 3c; Figure S24, Supporting Information), indicating activation of the pseudo‐inert basal planes. Material characterization confirmed that, compared to bulk‐KPHI, nano‐KPHI maintains high crystallinity and exhibits fewer defects (Figure S25, Supporting Information), with no significant differences in chemical structure or elemental composition. Therefore, the activation of basal planes in nano‐KPHI suggests the emergence of an additional mechanism that restricts long‐range exciton transport in nanosheets.

### 2.4 Exciton Self‐Trapping Phenomenon

**Figure 4:**
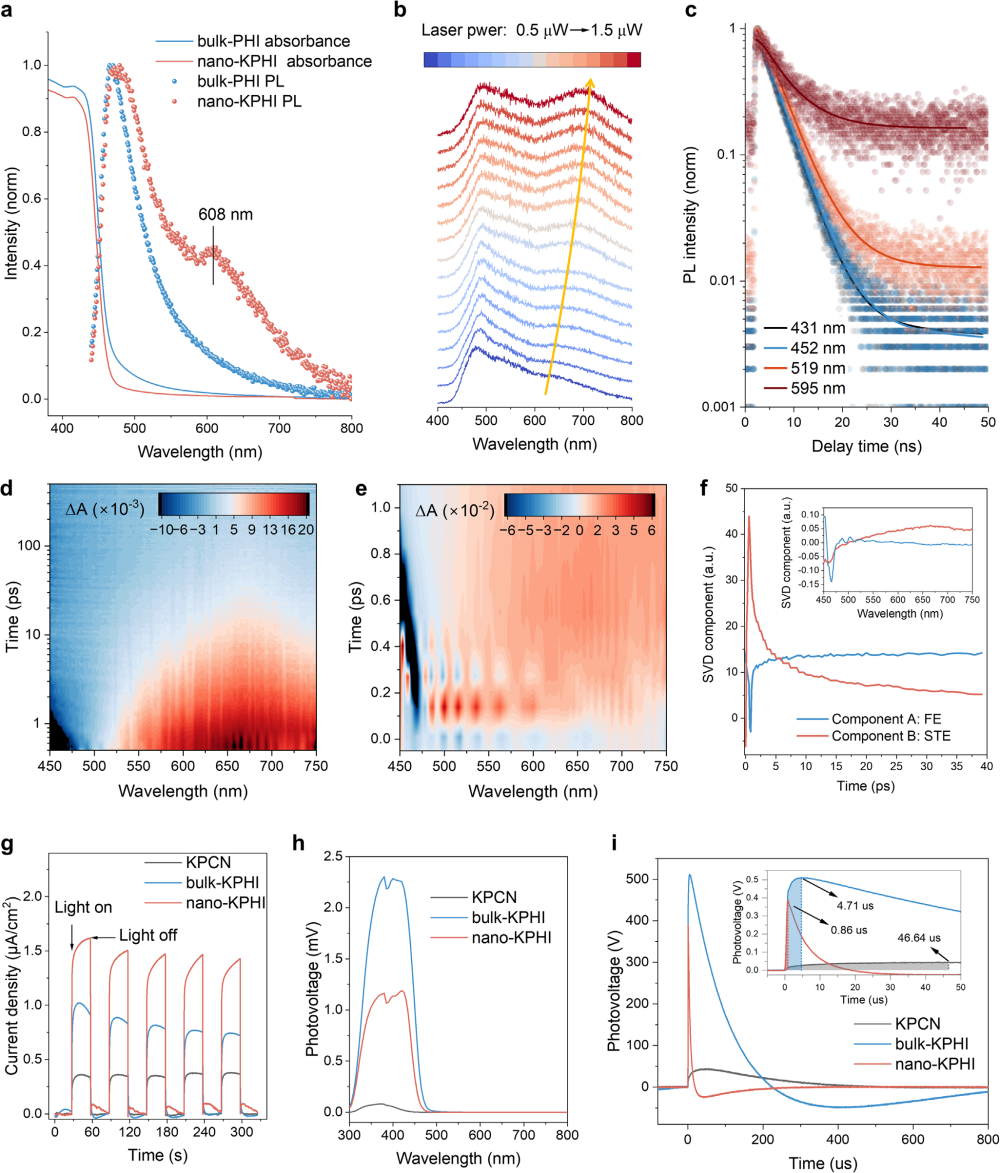
Photophysical characterization revealing exciton self‐trapping behavior. a) UV–vis absorption (lines) and fluorescence emission spectra (dots) of bulk‐KPHI (blue) and nano‐KPHI (red) at room temperature under 400 nm excitation. b) Power‐dependent PL spectra of nano‐KPHI showing spectral evolution from 0.5 µW (bottom, blue) to 1.5 µW (top, red). c) Time‐resolved fluorescence decay curves of nano‐KPHI monitored at 431 nm (dark blue), 452 nm (light blue), 519 nm (light red), and 595 nm (dark red). 2D mapping TA spectra of nano‐KPHI under 350 nm femtosecond laser excitation at room temperature in d) 0–1000 ps, and e) 0–1 ps. f) Associated kinetic components of nano‐KPHI were calculated by singular value decomposition analysis, with principle spectral components inset. g) Photocurrent responses, h) Steady‐state and i) TPV measurements of KPCN (gray), bulk‐KPHI (blue) and nano‐KPHI (red) under visible light illumination, with the charge extraction time, maximum number of charges shown inset.

To elucidate the mechanism restricting long‐range exciton transport in nano‐KPHI, we conducted UV–vis absorption and fluorescence emission spectroscopy on the prepared materials. Nano‐KPHI and bulk‐KPHI share similar absorption profiles, both showing maxima at 440 nm without observable sub‐band absorption. Compared with KPCN (Figure S26, Supporting Information), both materials exhibited a steeper decline in absorption intensity, indicating enhanced homogeneity and fewer structural defects (**Figure** **4**a). The emission characteristics, however, showed striking divergence. Bulk‐KPHI is dominated by a single and narrow emission band centred at 480 nm with minimal Stokes shift, characteristic of free exciton (FE) emission.^[^ ^27^ ^]^ In contrast, nano‐KPHI exhibited FE emission peak and an additional broad emission band at 608 nm with a substantial Stokes shift of 168 nm. These results indicate the presence of defect‐level emission within the band gap in nano‐KPHI, which may be key to limiting in‐plane exciton migration.

Material characterization confirmed that nano‐KPHI maintains high crystallinity without introducing additional structural defects compared to bulk‐KPHI. Thus, we attributed this unexpected emission behavior to light‐induced self‐trapped states (STS). In organic semiconductors, strong exciton‐phonon coupling can induce transient lattice distortions around photoexcited electron‐hole pairs, resulting in STS. These states can rapidly capture free excitons to form localized Frenkel‐like self‐trapped excitons (STEs).^[^ ^9^ ^]^ The emission characteristics of STEs include broad peaks with a width exceeding 60 nm and substantial Stokes shifts larger than 100 nm, consistent with our observations in nano‐KPHI.^[^ ^28^ ^]^ We propose that dimensional reduction through exfoliation lowers the lattice distortion energy barrier, thereby promoting STE formation.

To further verify the existence of STEs, we conducted power‐dependent photoluminescence (PL) measurements on nano‐KPHI (Figure 4b). The broad emission band showed a linear increase in intensity with increasing excitation power, accompanied by progressive red‐shifting. This behavior, distinct from defect‐related mid‐gap states, which typically show saturation at higher power levels, matches typical STE response where increased laser intensity enhances exciton trapping and amplifies lattice distortion, resulting in deeper self‐trapping energy levels.^[^ ^29^ ^]^ Notably, neither KPCN nor bulk‐KPHI exhibited such power‐dependent spectral evolution (Figure S27, Supporting Information), confirming that this phenomenon is unique to nano‐KPHI. In nano‐KPHI, time‐resolved photoluminescence measurements revealed wavelength‐dependent lifetime extension toward longer wavelengths (Figure 4c), which is consistent with the characteristically longer lifetime of STEs compared to FEs.^[^ ^30^ ^]^

Temperature‐dependent PL measurements were performed to investigate exciton behaviors. As shown in Figure S28 (Supporting Information), the overall PL intensity for nano‐KPHI increases with decreasing temperature due to suppressed nonradiative recombination.^[^ ^30^, ^31^ ^]^ The persistent presence of a broad STE emission peak whose intensity regularly increases as the temperature decreases indicates a dynamic equilibrium between exciton trapping and detrapping processes. An activation energy (E_a_) of ≈32.8 meV is extracted by fitting the integrated intensity with the Arrhenius equation (Figure S29, Supporting Information). Analysis of the temperature‐dependent linewidth of nano‐KPHI's STE emission revealed a large Huang–Rhys factor (*S* = 29.9) and phonon energy (E_phonon_ = 26.4 meV) with an estimated STE formation time of 157 fs (fitting results in Figure S30, Supporting Information).^[^ ^32^ ^]^ A large S indicates strong exciton‐phonon coupling, a prerequisite for STE.^[^ ^33^ ^]^ The measured E_phonon_ is consistent with typical low‐frequency vibrations of 2D materials,^[^ ^34^ ^]^ suggesting STE formation involves in‐plane shear or out‐of‐plane breathing vibration primarily driven by strong exciton‐phonon coupling to these modes, forming the self‐trapped potential well.

Femtosecond transient absorption (fs‐TA) spectroscopy provides direct insight into exciton self‐trapping dynamics. Following 350 nm pump pulse excitation, both bulk‐KPHI (Figure S31, Supporting Information) and nano‐KPHI (Figure 4d) showed a prominent bleaching band at 450 nm.^[^ ^35^ ^]^ In the 520–750 nm range, nano‐KPHI exhibited a broad positive photoinduced absorption (PIA) characteristic of STEs,^[^ ^28a^ ^]^ whereas bulk‐KPHI showed minimal absorption, suggesting negligible STE formation.

To gain deeper insight into the mechanism and timescale of STE formation, we performed a detailed analysis of the TA spectra within the initial picosecond following excitation. The 2D TA mapping of nano‐KPHI reveals clear spectral evolution with increasing delay time, indicating complex photophysical processes occurring immediately after photoexcitation (Figure 4e). In the subsequent 140–270 fs timeframe after excitation, a broad positive PIA feature emerges, spanning from ≈500 to 700 nm (Figure S32, Supporting Information). This spectral signature is characteristic of self‐trapped exciton formation,^[^ ^36^ ^]^ and the observed timescale aligns well with STE formation time (174 fs) yield by temperature‐dependent PL measurements and previous literature reports (<200 fs).^[^ ^28a^ ^]^ Within 500 fs post‐excitation, we observe the development of additional positive induced absorption signals in the 475–600 nm region. These features likely correspond to intermediate processes in the exciton trapping pathway, possibly involving sequential localization of photogenerated carriers into self‐trapped states.^[^ ^32^, ^36^, ^37^ ^]^

Singular value decomposition (SVD) was employed to analyze the complex femtosecond TA results for comprehensive understanding of excitation dynamics. As shown in Figure 4f, the TA data of nano‐KPHI can be well described by two principal components (A and B). Component A is dominated by ground‐state bleaching peaks with an average lifetime of 880 fs and can be attributed to free excitons. Component B represents PIA superimposed on the ground state bleaching signal, with an average lifetime of 7.02 ps, which can be assigned to STEs. These results suggest that FEs and STEs form simultaneously upon excitation and reach their maximum values at 0.83 and 0.54 ps, respectively, before beginning to decay. The fitted decay dynamics of FEs and STEs are summarized in Table S1 (Supporting Information).

### 2.5 Charge Separation and Transfer Properties

Having observed the presence of reversible exciton self‐trapping in nano‐KPHI, we next investigated how this mechanism influences interfacial charge separation and transfer. Photocurrent measurements revealed a sequential increase from KPCN to bulk‐KPHI to nano‐KPHI (Figure 4g). Paradoxically, steady–state photovoltage measurements showed that bulk‐KPHI generated significantly stronger signals than nano‐KPHI (Figure 4h). To resolve this apparent contradiction, we conducted transient photovoltage (TPV) measurements, and the quantitative analysis revealed distinct charge dynamics for each material (Table S1, Supporting Information).^[^ ^38^ ^]^ All KPHI samples exhibited efficient internal exciton transport compared to KPCN, as evidenced by the rapid signal generation times of 4.76 and 0.86 µs for bulk‐KPHI and nano‐KPHI, respectively, significantly faster than 46.64 µs for KPCN (Figure 4i). However, further analysis showed contrasting charge accumulation and transfer behaviors. Nano‐KPHI exhibited a smaller charge decay constant and a lower effective charge density on its surface compared to bulk‐KPHI (Figure S33, Supporting Information).

This divergent charge transfer behavior between bulk and nano‐KPHI can be understood through the interplay of exciton dynamics, structural features, and dimensional effects. In bulk‐KPHI, despite efficient charge generation, the migration of excitons toward edges leads to accumulation at trapping sites with dangling bonds, hindering efficient interfacial charge transfer as indicated by the prolonged relaxation time.^[^ ^39^ ^]^ Conversely, in nano‐KPHI, both STE‐induced exciton localization on basal planes and shorter transport pathways due to reduced dimensions contribute to faster charge transfer. Together, these effects result in lower steady–state signals but accelerated relaxation kinetics. Additionally, nano‐KPHI presented a lower photoluminescence intensity (Figure S34, Supporting Information) and reduced charge transfer resistance (Figure S35, Supporting Information). This finding demonstrates that STE formation can cooperate with dimensional effects to enhance interfacial charge transfer in photocatalysis.^[^ ^40^ ^]^

### 2.6 Photo‐Driven H_2_O_2_ Production

**Figure 5:**
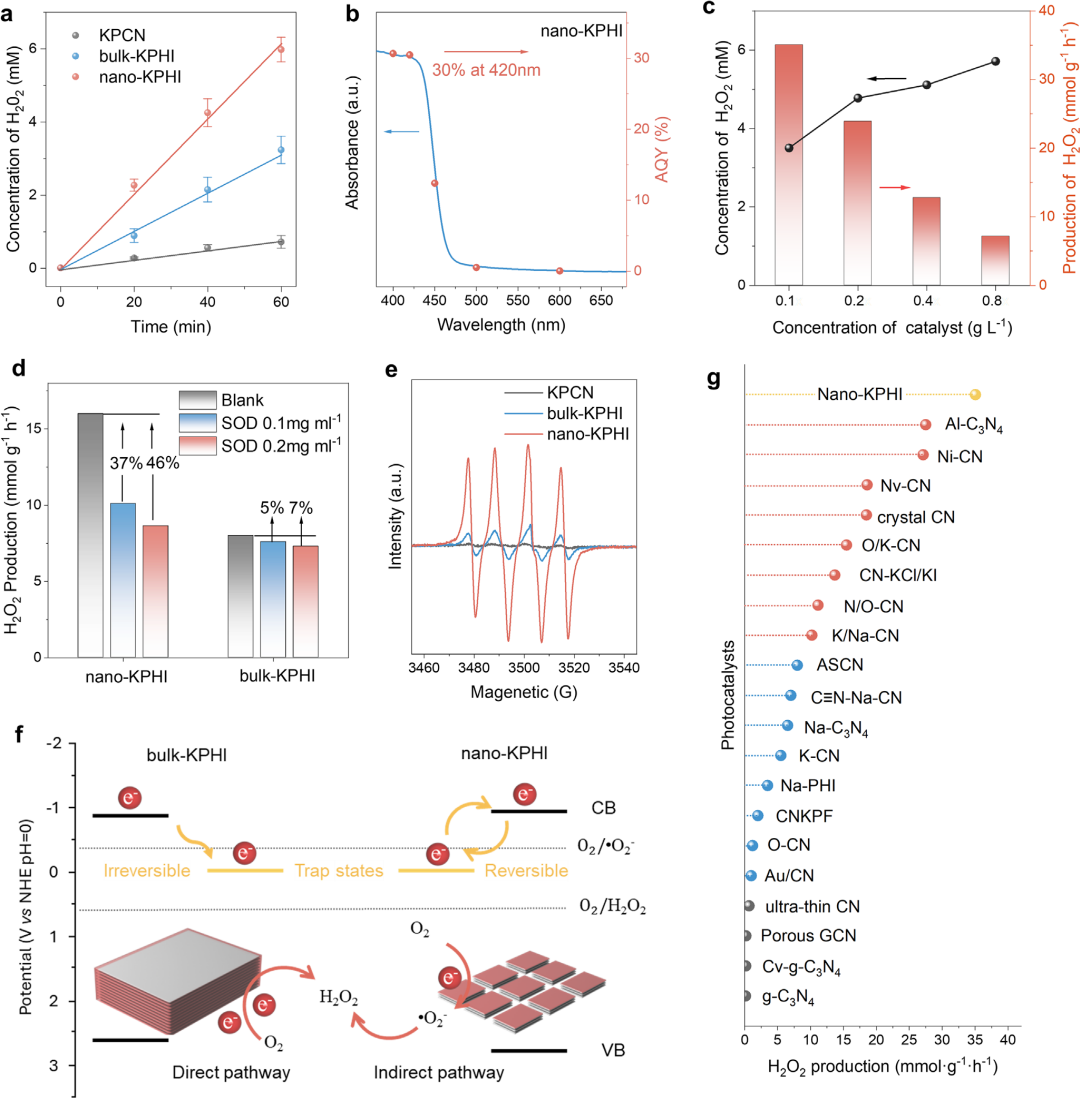
Hydrogen peroxide generation. a) Time course of H_2_O_2_ production measured under visible light irradiation (λ ≥ 380 nm, 300 mW cm^−2^; 15 mg catalyst in 30 ml 10% ethanol solution, 0.5 g L^−1^ photocatalyst). b) AQY and corresponding UV–vis DRS of H_2_O_2_ production by nano‐KPHI under monochromatic light irradiation of different wavelengths. c) H_2_O_2_ production rate of nano‐KPHI at varying catalyst loadings. d) Photocatalytic H_2_O_2_ generation rate with the addition of SOD (λ ≥ 380 nm, 300 mW cm^−2^; 10 mg catalyst in 10 ml 10% ethanol solution, 1 g L^−1^ photocatalyst). e) EPR signals of DMPO‐•O_2_^−^ over KPCN, bulk‐KPHI, and nano‐KPHI. f) Schematic illustration of how edge defects and self‐trapping lead to distinct H_2_O_2_ production pathways. g) Comparison with other CN‐based photocatalysts in sacrificial agent systems from recent works for H_2_O_2_ production.

The importance of activating inert basal planes was demonstrated in the photocatalytic oxygen reduction to H_2_O_2_, an important chemical in industry.^[^ ^1^, ^41^ ^]^ The reaction was conducted using the catalyst dispersed in a 10 v% ethanol solution with photocatalyst dosage of 0.5 g L^−1^, under visible light irradiation (λ ≥ 380 nm, 300 mW cm^−2^) in an O_2_ atmosphere. As shown in **Figure** **5**a, nano‐KPHI achieved a significantly higher H_2_O_2_ rate of 12 mmol g^−1^ h^−1^, which is eight times greater than that of bulk‐KPCN (1.4 mmol g^−1^ h^−1^) and twice that of bulk‐KPHI (6.4 mmol g^−1^ h^−1^). The apparent quantum yield (AQY) of nano‐KPHI was closely correlated with its absorption spectrum and demonstrated wavelength‐dependent photocatalytic activity, with an AQY as high as 30% at 420 nm (Figure 5b). Stability tests showed that nano‐KPHI maintained consistent performance over five cycles (Figure S36, Supporting Information). Notably, the nanosheet morphology of nano‐KPHI significantly enhanced its catalytic performance even at reduced catalyst loadings, owing to its increased specific surface area (Figure S37, Supporting Information), and superior dispersibility in reaction media (Figure S38, Supporting Information). Reducing catalyst concentration from 0.8 to 0.1 mg mL^−1^ led to less than 50% decrease in H_2_O_2_ concentration generated over 1 h (Figure 5c). However, the normalized production rate increased significantly, increasing from 7 to 35 mmol g^−1^ h^−1^. In contrast, at the same catalyst concentration, bulk‐KPHI only achieved 7.7 mmol g^−1^ h^−1^ (Figure S39, Supporting Information).

Considering that the relocation of the reactive activity centers of nano‐KPHI and bulk‐KPHI upon activation of the inert basal planes may cause changes in the H_2_O_2_ generation mechanism, we have explored the reaction pathway of photocatalytic H_2_O_2_ generation by the bulk and nano‐KPHI samples. As shown in Figure S40 (Supporting Information), no H_2_O_2_ generation was observed under light‐free conditions, which confirmed that the process was photochemically driven. Furthermore, H_2_O_2_ formation was inhibited under N_2_ atmosphere and in the presence of Ag^+^ (as an electron acceptor), confirming that the oxygen reduction reaction (ORR) is the primary mechanism.

Previous studies have indicated that the ORR of H_2_O_2_ production can proceed via direct and indirect two‐electron pathways.^[^ ^42^ ^]^ In the indirect pathway, O_2_ first receives a photogenerated electron to generate a superoxide anion radical, which subsequently receives an electron and a proton to be reduced to H_2_O_2_. The direct ORR pathway has a more positive reaction potential (+0.68 V) and is preferred thermodynamically, but the indirect ORR pathway is more prone to favor kinetic considerations because it involves a single‐electron step.^[^ ^43^ ^]^ To determine the predominant pathway for each catalyst, we first analyzed their band structures. The conduction band potentials of KPCN, bulk‐KPHI, and nano‐KPHI were determined to be −0.82, −0.81, and −0.81 V (vs. RHE) from Mott–Schottky analysis (Figure S41, Supporting Information), indicating that both direct and indirect pathways are thermodynamically feasible (Figure S42, Supporting Information).

To determine the H_2_O_2_ generation pathway for each catalyst, we added superoxide radical dismutase (SOD), a scavenger for superoxide radical(•O_2_^−^), to the reaction system, which can reduce the efficiency of the indirect two‐electron pathway of H_2_O_2_ generation (with a theoretical maximum reduction of 50%).^[^ ^44^ ^]^ The results showed that the rate of H_2_O_2_ generation by nano‐KPHI decreased by 37% in the presence of 0.1 mg mL^−1^ SOD and by 46%, which is close to the theoretical maximum (Figure 5d), when the SOD concentration was increased to 0.2 mg mL^−1^. In contrast, bulk‐KPHI was less affected by SOD, and the H_2_O_2_ generation rate only decreased by only 5% and 7%, respectively. Furthermore, nano‐KPHI demonstrated stronger •O_2_^−^ spin trapping signals in the electron paramagnetic resonance (EPR) spectra compared to bulk‐KPHI (Figure 5e). These results indicate that, despite having similar band positions, nano‐KPHI predominantly generates H_2_O_2_ through the indirect pathway, while bulk‐KPHI follows the direct pathway.

To further investigate the reactive species involved in the photocatalytic process, comprehensive EPR measurements were conducted using specific spin traps.^[^ ^45^ ^]^ No significant signals for hydroxyl radicals (•OH) were detected (Figure S43, Supporting Information), which is consistent with H_2_O_2_ production primarily occurring via ORR pathways rather than those involving water oxidation. EPR results confirmed the generation of singlet oxygen (^1^O_2_) in the reaction system. Notably, the signal intensity for ^1^O_2_ was significantly higher for nano‐KPHI compared to bulk‐KPHI and KPCN, and it increased with illumination time, indicating its photocatalytic generation (Figure S44, Supporting Information). However, control experiments utilizing sodium azide as a selective ^1^O_2_ scavenger showed that while the EPR signal of ^1^O_2_ was suppressed, the production of H_2_O_2_ was not inhibited (Figure S45, Supporting Information). This finding demonstrates that, despite being generated, ^1^O_2_ is not an intermediate in the primary H_2_O_2_ formation pathway under our reaction conditions. Based on further experiments, including scavenging studies with SOD, the generation of ^1^O_2_ appears to originate from •O_2_^−^ via an electron‐hole surface recombination process mediated by oxygen.^[^ ^46^ ^]^

As conceptually illustrated in Figure 5f, we propose that in bulk‐KPHI, excitons migrating to edge sites encounter structural defects such as terminal groups and dangling bonds. These defects cause irreversible trapping and energy loss, rendering the indirect pathway, which requires a more negative potential, unfavorable. In contrast, the reversible nature of STE in nano‐KPHI preserves energy levels, enabling H_2_O_2_ production through the indirect pathway. The significantly higher ^1^O_2_ yield observed on nano‐KPHI, while a side reaction, serves as compelling additional evidence for the enhanced availability of excitons on the activated basal planes. Therefore, the superior performance of nano‐KPHI can be attributed to the advantages of its basal‐plane activity, including fast interfacial electron transfer and the kinetic benefits of the indirect two‐electron pathway. Compared with previously reported carbon nitride‐based catalysts (Figure 5g; Table S3, Supporting Information), nano‐KPHI demonstrated a remarkably high yield for photocatalytic H_2_O_2_ production.

These results demonstrate that basal plane activation in 2D organic semiconductors not only dramatically enhances photocatalytic performance but fundamentally alters reaction pathways, highlighting the importance of correctly identifying and addressing pseudo‐inert basal planes in future research. This pseudo‐inertness originates from the characteristic electronic structure of 2D organic semiconductors, specifically the continuous in‐plane covalent bonding coupled with weak van der Waals interactions between layers.^[^ ^14^ ^]^ This structural feature leads to anisotropic exciton migration preferentially toward edges rather than across basal planes, as demonstrated in our study. 2D organic semiconductors with extended π‐electron systems, in‐plane ionic doping, or highly ordered lattices likely exhibit enhanced anisotropic exciton transport and may therefore encounter similar pseudo‐inert basal plane challenges. The exciton self‐trapping may provide a generalizable solution to this issue in other 2D materials, as they have similar electronic characteristics and anisotropic transport of charge carriers. It is known that soft lattices and reduced structural dimensionality are generally considered prerequisites for achieving self‐trapping.^[^ ^9^ ^]^ Organic semiconductors, with their discontinuous conjugated systems, inherently possess soft lattices.^[^ ^8a^ ^]^ As demonstrated in this work, the exfoliation method for dimensional reduction could also be able to induce self‐trapping in other 2D organic semiconductors. As such, we believe that exciton self‐trapping induced by reduced dimensionality would provide a broadly applicable strategy to activate the pseudo‐inert basal planes of 2D organic semiconductors.

## 3 Conclusion

In this study, we identify “pseudo‐inert” basal planes as a previously unrecognized challenge in crystalline 2D organic photocatalysts and present a strategic approach to overcome this limitation. In disordered photocatalysts (amorphous or semicrystalline), basal planes contribute to apparent catalytic activity; however, their structural disorder promotes exciton localization and accelerates bulk recombination, compromising overall efficiency. Conversely, in highly crystalline 2D frameworks, efficient long‐range exciton transport to edge sites prevents bulk recombination but renders intrinsically active basal planes pseudo‐inert. This spatial distribution of reactive sites depends on the characteristic electronic structure of 2D organic photocatalysts and is therefore likely to be a universal phenomenon. We demonstrate that controlled dimensional reduction induces reversible exciton self‐trapping, effectively reactivating these pseudo‐inert basal planes while preserving crystalline order and minimizing recombination losses. The efficacy of this approach is quantitatively validated by the exceptional H_2_O_2_ production rate of 35 mmol g^−1^ h^−1^ achieved with nano‐KPHI. Mechanistic studies reveal that activated basal planes and edge sites facilitate H_2_O_2_ generation via distinct reaction pathways, emphasizing the necessity of correctly identifying and regulating basal plane activity in 2D organic photocatalysts. Collectively, these insights provide new design principles for high‐performance 2D organic photocatalysts and suggest a general strategy to address pseudo‐inert surface challenges in other excitonic photocatalytic systems.

## Conflict of Interest

The authors declare no conflict of interest.

## Supporting information



Supporting Information

## Data Availability

The data that support the findings of this study are available from the corresponding author upon reasonable request.
