# Supplementary material for: Self‐Trapped Excitons Activate Pseudo‐Inert Basal Planes of 2D Organic Semiconductors for Improved Photocatalysis
Source: Adv Mater. 2025 May 16;37(30):2505653. doi: 10.1002/adma.202505653 (PMC12306381; doi:10.1002/adma.202505653)
Supplement: Supplementary file 1 — Supporting Information [file ADMA-37-2505653-s001.pdf]

ADVANCED MATERIALS

Supporting Information

for *Adv. Mater.*, DOI 10.1002/adma.202505653

Self-Trapped Excitons Activate Pseudo-Inert Basal Planes of 2D Organic Semiconductors for Improved Photocatalysis

Jindi Yang, Xiangkang Zeng, Bicheng Zhu, Sharidya Rahman, Chuanbiao Bie, Ming Yong, Kaige Sun, Mike Tebyetekerwa, Zhuyuan Wang, Lijun Guo, Xin Sun, Yuan Kang, Lars Thomsen, Zhimeng Sun, Zhongguo Zhang* and Xiwang Zhang**

Supporting Information

Self-trapped excitons activate pseudo-inert basal planes of 2D organic semiconductors for improved photocatalysis

Jindi Yang[1], Xiangkang Zeng[1], Bicheng Zhu[2], Sharidya Rahman[3,4] Chuanbiao Bie[2], Ming Yong[1,5], Kaige Sun[1], Mike Tebyetekerwa[1], Zhuyuan Wang[1], Lijun Guo[1], Xin Sun[1], Yuan Kang[5], Lars Thomsen[6], Zhimeng Sun[7], Zhongguo Zhang[7]*, and Xiwang Zhang[1,8]**

[1]Dow Centre for Sustainable Engineering Innovation, School of Chemical Engineering, The University of Queensland St Lucia, QLD 4072, Australia

[2]Laboratory of Solar Fuel, Faculty of Materials Science and Chemistry, China University of Geosciences, 68 Jincheng Street, Wuhan 430078, P. R. China

[3]ARC Centre of Excellence in Exciton Science

[4] Department of Materials Science & Engineering, Monash University, Clayton, VIC 3800, Australia

[5]Department of Chemical and Biological Engineering, Monash University, Clayton, VIC 3800, Australia

[6] Australian Synchrotron, 800 Blackburn Road, Clayton, 3168, Victoria, Australia

[7]Institute of Resource and Environment, Beijing Academy of Science and Technology, North Xisanhuan Road 27, Haidian District, Beijing 100089, China

[8]ARC Centre of Excellence for Green Electrochemical Transformation of Carbon Dioxide, The University of Queensland St Lucia, QLD 4072, Australia

**** Correspondence***: x.zeng@uq.edu.au, zhangzhongguo@irebjast.ac.cn, xiwang.zhang@uq.edu.au

Experimental Section

Chemicals

All chemicals were used directly without additional purification. Melamine ($C_3H_6N_6$, 99.0%), potassium chloride (KCl, 99.0%), and ethanol (EtOH, 99.0%) were procured from Aladdin Biochemical Technology Co. Ltd., China. Superoxide dismutase from bovine erythrocytes (SOD, > 3000 units/mg protein), potassium hexachloroplatinate (K_2PtCl_6, 98%), N,N-diethyl-p-phenylenediamine (DPD, 97%) and horseradish peroxidase (POD, RZ > 1.5) were obtained from Sigma-Aldrich. Milli-Q water served as the solvent in all experiments.

Preparation of Photocatalysts

A mixture of melamine (5.0 g) and KCl (15 g) was dispersed in ethanol (30 mL) and homogenized for 30 min. After complete ethanol evaporation at 80°C, the resulting mixtures were transferred to a covered 50 mL crucible and calcined in a muffle furnace under ambient air conditions. The samples were heated at a rate of 5°C min^{-1} to final temperatures of 550°C, 575°C, and 600°C for 2 h each, to obtain KPCN, bulk-KPHI, and nano-KPHI, respectively. The products were mechanically ground, thoroughly washed with deionized water, and dried overnight at 80°C before further use.

Photo-deposition Experiments

Photodeposition experiments were conducted to investigate the spatial distribution of surface reactive centres. The photocatalyst (10 mg) was dispersed in 20 mL of 10% (v/v) aqueous ethanol solution. Dissolved oxygen was removed by purging with N_2 for 30 min under dark conditions. Subsequently, $H_2PtCl_6 \cdot 6H_2O$ was added to achieve a nominal Pt loading of 1 wt%. Ethanol served as a hole scavenger, enabling the photoreduction of Pt^{4+} ions to Pt nanoparticles, which acted as markers for catalytically active sites[1].

Computational details

Density functional theory (DFT) calculations were performed using the Vienna Ab initio Simulation Package (VASP). The exchange-correlation interactions were described using the generalized gradient approximation (GGA) with the Perdew-Burke-Ernzerhof (PBE) functional. The plane-wave energy cutoff was set to 450 eV. Geometry optimizations were conducted with convergence criteria of 10^{-5} eV for total energy and 0.02 eV $Å^{-1}$ for forces. Surface models were constructed with a 15 Å vacuum layer

to eliminate spurious interactions between periodic images.

Characterizations

Powder X-ray diffraction (XRD) analysis was conducted in the range of 5-90 degrees using a diffractometer equipped with Cu Kα radiation (BRUKER, D2 PHASER). Fourier transform infrared spectroscopy (FT-IR) was obtained using a spectrometer (Thermo Scientific Nicolet iS20). X-ray photoelectron spectroscopy (XPS) measurements were performed using a Thermo Scientific K-Alpha XPS spectrometer. Solid-state nuclear magnetic resonance (NMR) spectra of 13C, 15N, and 1H nuclei were recorded on a BRUKER AVANCE NEO 400WB instrument. The optical properties of the samples were analysed using a UV-vis spectrophotometer (Shimadzu UV-3600i Plus). Steady-state photoluminescence (PL) spectra for quantitative comparison were collected at room temperature on an Edinburgh FLS100 fluorescence spectrophotometer with a 400 nm excitation wavelength. Power-dependant photoluminescence measurements were conducted using Confocal Microscope System (Witec Alpha 300R) with a 10x objective lens (NA = 0.45) in ambient condition. A fibre coupled 355 nm CW laser having a spot size of 1 μm diameter was used as excitation source. The samples were illuminated from the top side on a piezo-crystal-controlled scanning stage. Near-Edge X-ray Absorption Fine-Structure (NEXAFS) Spectroscopy measurements were performed on the Soft X-ray beamline at the Australian Synchrotron[2]. An X-ray incident angle of 55° (known as the "magical angle") was used for all spectra and Total Electron Yield (TEY) signals were collected. The recorded signals were normalised by the "stable monitor method" and by setting the pre-edge to 0 and the post-edge to 1.[3] The collected data were analysed using the Quick AS NEXAFS Tool (QANT) macro package implemented in IgorPro (8.04).[4]

Photoelectrochemical measurements

The photoelectric properties were assessed using a standard three-electrode system coupled with a CHI760E electrochemical workstation. A saturated Ag/AgCl electrode served as the reference electrode, while a platinum wire acted as the counter electrode. The working electrode consisted of a FTO conductive glass coated with the photocatalyst. To prepare the working electrode, a catalyst slurry (150 μL) was evenly coated onto the FTO substrate, which had an effective working area of 0.8 cm^2. Subsequently, the coated electrode was dried at room temperature. A 500 W Xe lamp (CEL-S500F,

Beijing CEAULight Co., China) equipped with a cutoff filter at 420 nm was employed as the light source. Transient photocurrent curves were recorded under a bias voltage of 0.5 V, with a 0.5 M Na_2SO_4 aqueous solution serving as the electrolyte. Mott-Schottky (MS) curves were generated by applying an AC voltage with frequencies of 1500, 2000, and 2500 Hz, and an applied voltage range of -1 to 1 V, in a 0.5 M Na_2SO_4 solution. Electrochemical impedance spectroscopy (EIS) was performed by applying an AC voltage with an amplitude of 5 mV in the frequency range from 10^5 to 1 Hz, with a 0.5 M Na_2SO_4 aqueous solution used as the electrolyte. Transient and steady-state surface photovoltage (SPV) tests were conducted on a surface photovoltage spectrometer (CEL-SPS1000, Beijing CEAULight Co., China).

Photocatalytic H_2O_2 production

Photocatalytic H_2O_2 production was evaluated in a 30 mL O_2-saturated aqueous solution containing 10 vol% ethanol as a hole scavenger and 0.5 g L^{-1} catalyst. The reaction was conducted under visible light irradiation ($\lambda \geq 380$ nm, Xenon lamp, PLS-SXE300/300UV, Beijing Perfectlight) with continuous O_2 bubbling at 0.8 mL min^{-1}.

Hydrogen Peroxide Concentration Measurement

The concentration of H_2O_2 was determined using the DPD/POD colorimetric method (**Figure S1**)[5]. Specifically, 30 uL of a 10 mg/mL DPD solution and 30 uL of a 1 mg/mL POD solution were mixed with 3 mL of a phosphate buffer (pH 6.48). Then, 1 mL of the appropriately diluted sample solution was added to the mixture and shaken for 90 seconds to allow the color development reaction to proceed. The absorbance of the clear solution was measured at 551 nm using a UV-visible spectrophotometer (**Figure S2**). The stock solutions were prepared as follows: DPD solution was made by dissolving 50 mg of DPD in 5 mL of 0.05 M H_2SO_4, while the POD solution was prepared by dissolving 5 mg of POD in 5 mL of ultrapure water. The phosphate buffer solution was created by combining 87.7 mL of 1 M $NaH_2PO_4 \cdot 2H_2O$ and 12.6 mL of 1 M $Na_2HPO_4 \cdot 12H_2O$ in 99.7 mL of water.

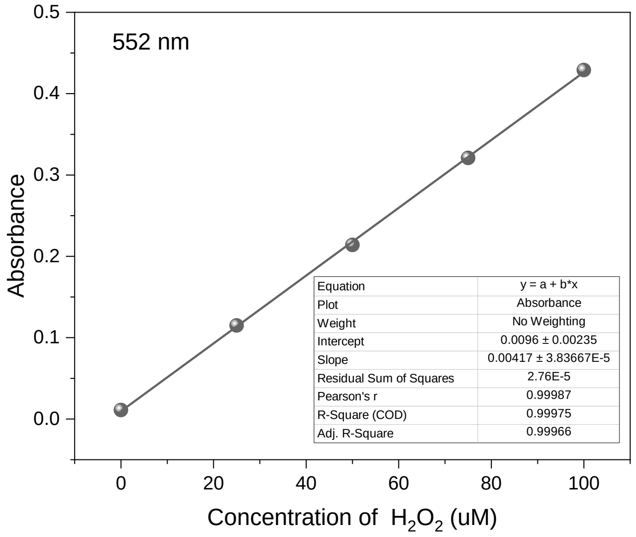

Figure S1. The linear relationship of concentration of H_2O_2 vs. UV-vis absorption intensity.

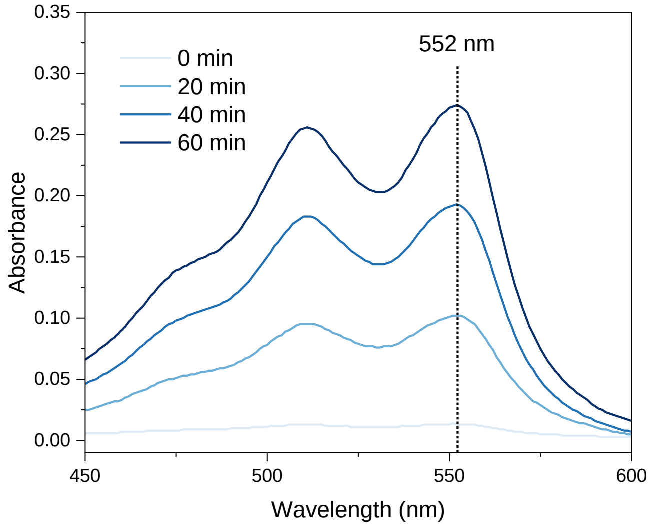

Figure S2. Time-dependent UV–vis absorption spectra of H_2O_2 detected by DPD method for nano-KPHI at a dilution ratio of 100.

H_2O_2 production pathway investigation

Mechanistic studies of H_2O_2 production were conducted through control experiments under various conditions: light exclusion, oxygen-free environment (N_2 purging), addition of $AgNO_3$ (0.1 M), and superoxide dismutase (SOD) at concentrations of 0.1 and 0.2 mg mL^{-1}. Radical species were analyzed

using a Bruker EMXplus-6/1 electron paramagnetic resonance (EPR) spectrometer. For spin trapping experiments, 5,5-dimethyl-1-pyrrolidine N-oxide (DMPO, 10 mM) was employed as the spin trap. The detection of hydroperoxyl radicals (*OOH) was performed using DMPO in methanol. Photocatalytic reactions were initiated using a 300 W Xenon lamp equipped with a 380 nm cutoff filter as the illumination source.

Supporting Figures

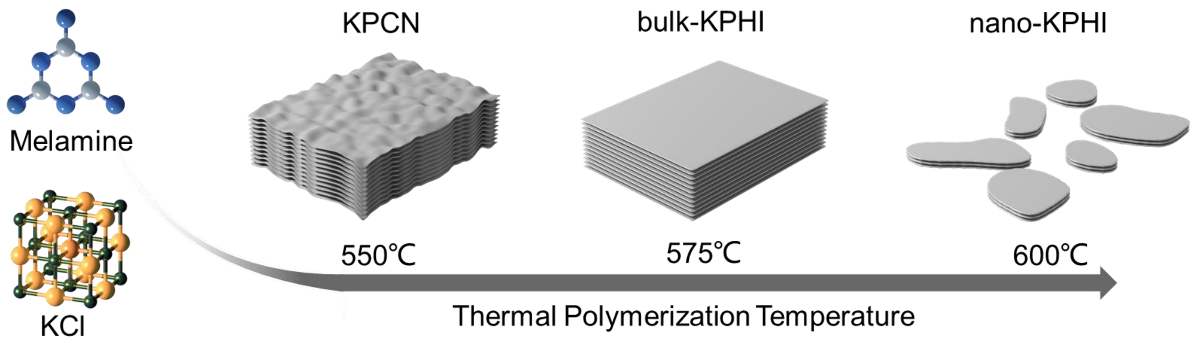

Figure S3. Schematic illustration of the temperature-controlled synthesis strategy. Melamine and KCl are thoroughly mixed using a homogenizer and then thermally treated in a muffle furnace. At 550°C, the reaction yields amorphous KPCN. Increasing the temperature to 575°C triggers crystallization to form bulk-KPHI. Further elevation to 600°C induces controlled fragmentation, producing nano-KPHI during subsequent aqueous processing.

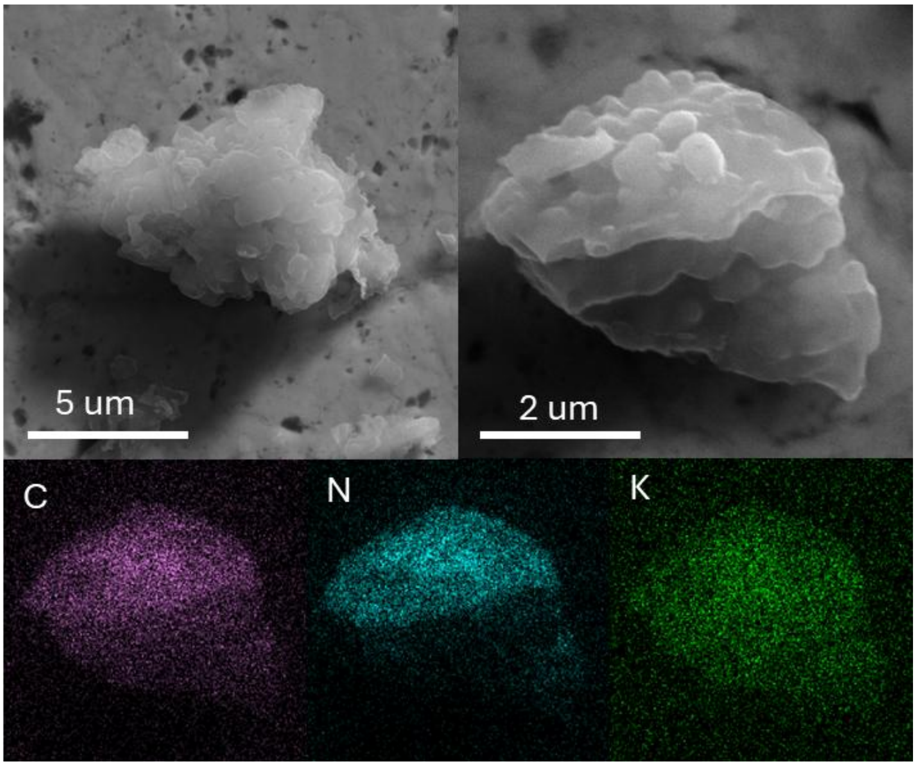

Figure S4. SEM and EDS mapping image of KPCN.

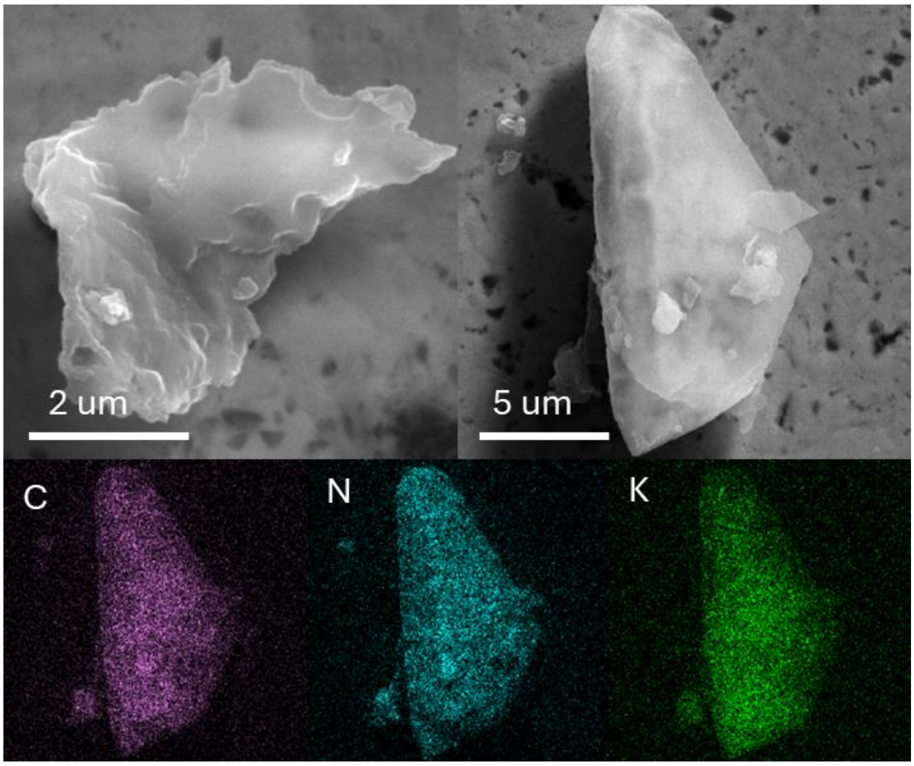

Figure S5. SEM and EDS mapping image of bulk-KPHI.

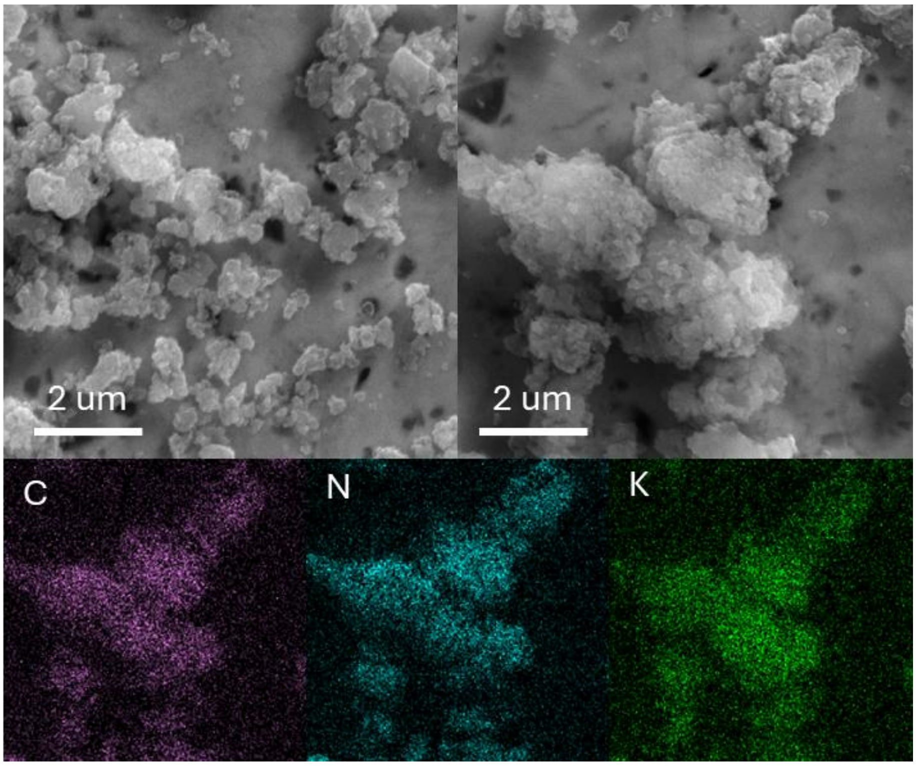

Figure S6. SEM and EDS mapping image of nano-KPHI.

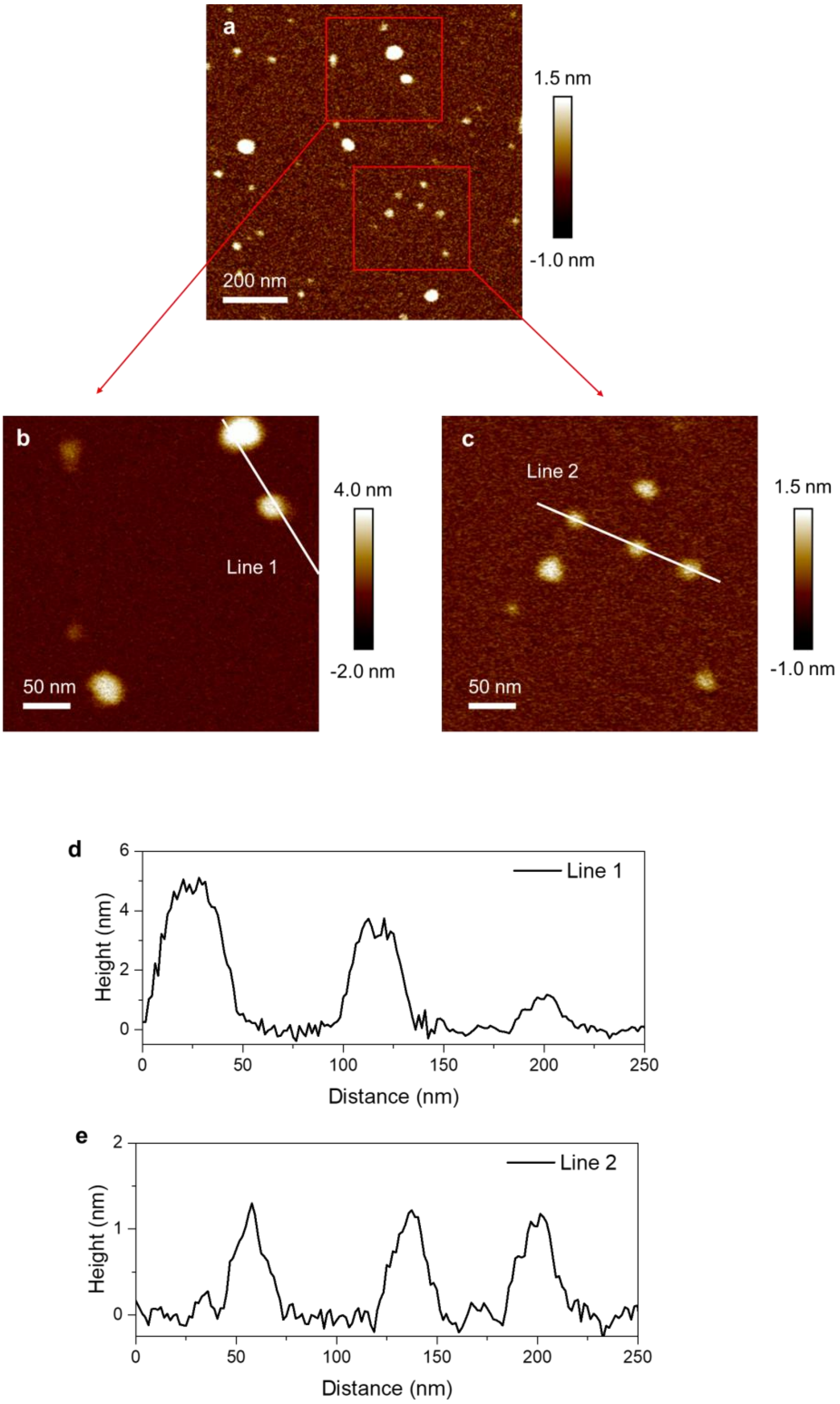

Figure S7. AFM characterization of nano-KPHI. a) b) and c) AFM images for statistical analysis of nano-KPHI. d) and e) The corresponding height profiles along the lines in the images.

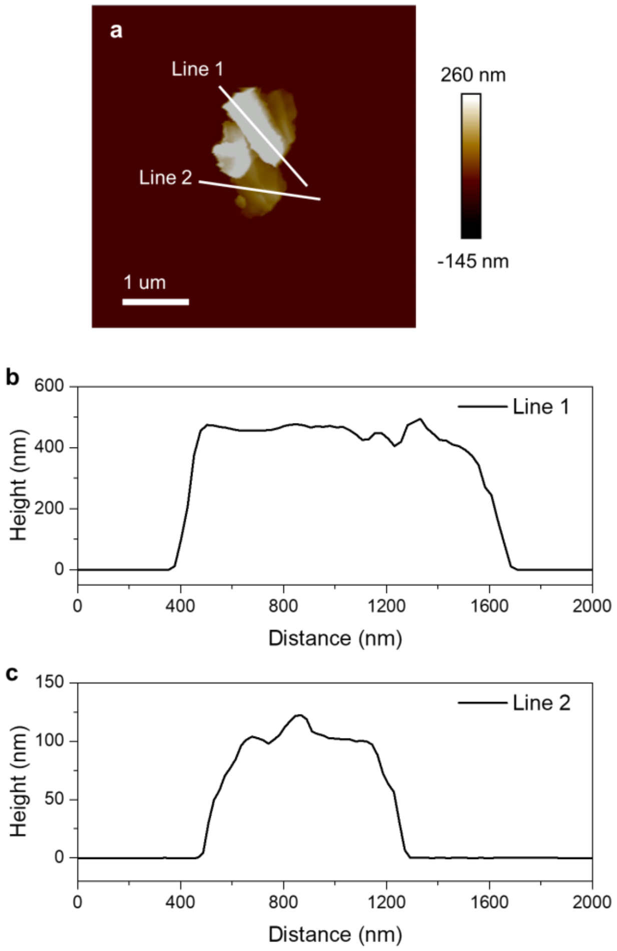

Figure S8. AFM characterization of bulk-KPHI. a) AFM images for statistical analysis of bulk-KPHI. b) and c) The corresponding height profiles along the lines in the images.

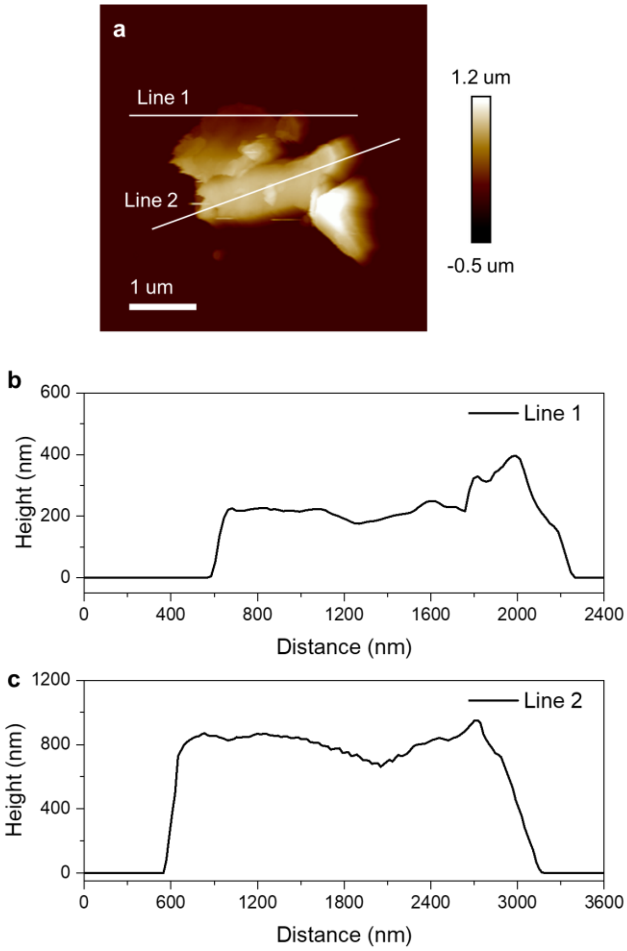

Figure S9. AFM characterization of KPCN. a) AFM images for statistical analysis of KPCN. b) and c) The corresponding height profiles along the lines in the images.

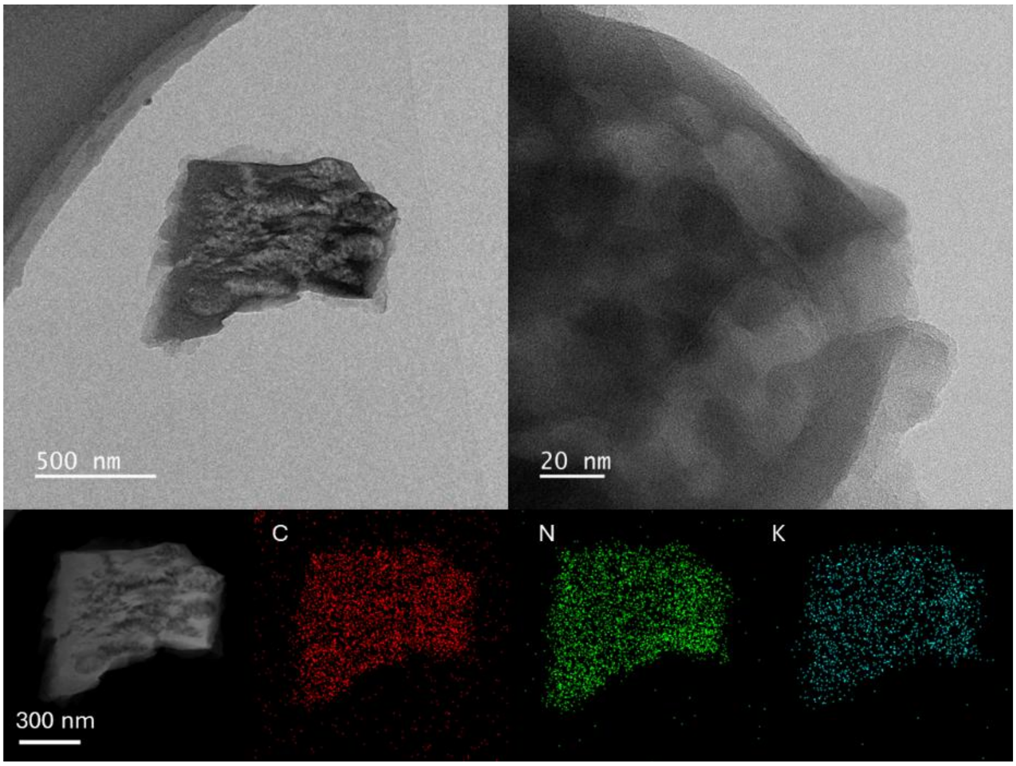

Figure S10. TEM and EDS mapping images of KPCN.

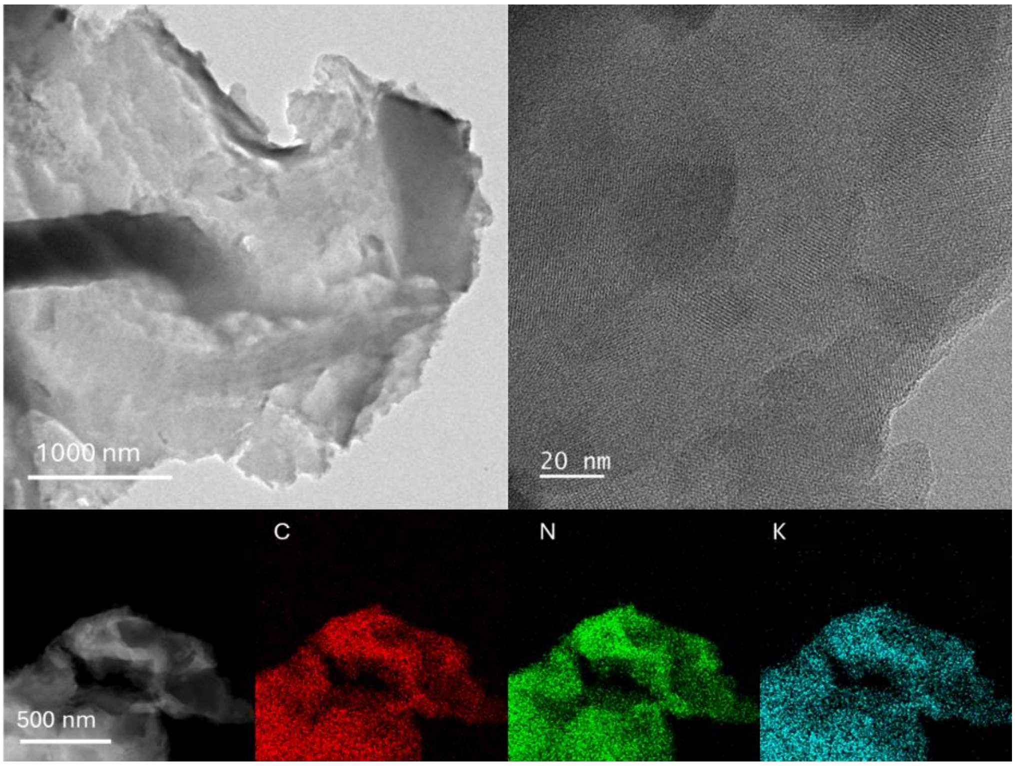

Figure S11. TEM and EDS mapping images of bulk-KPHI.

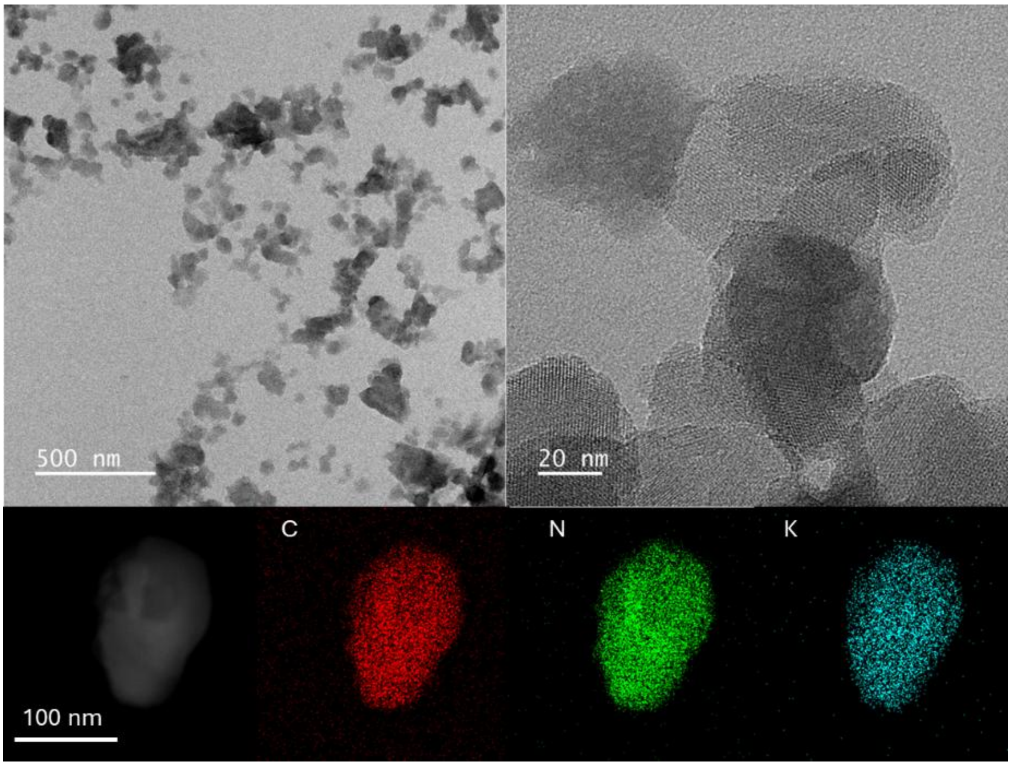

Figure S12. TEM and EDS mapping images of nano-KPHI.

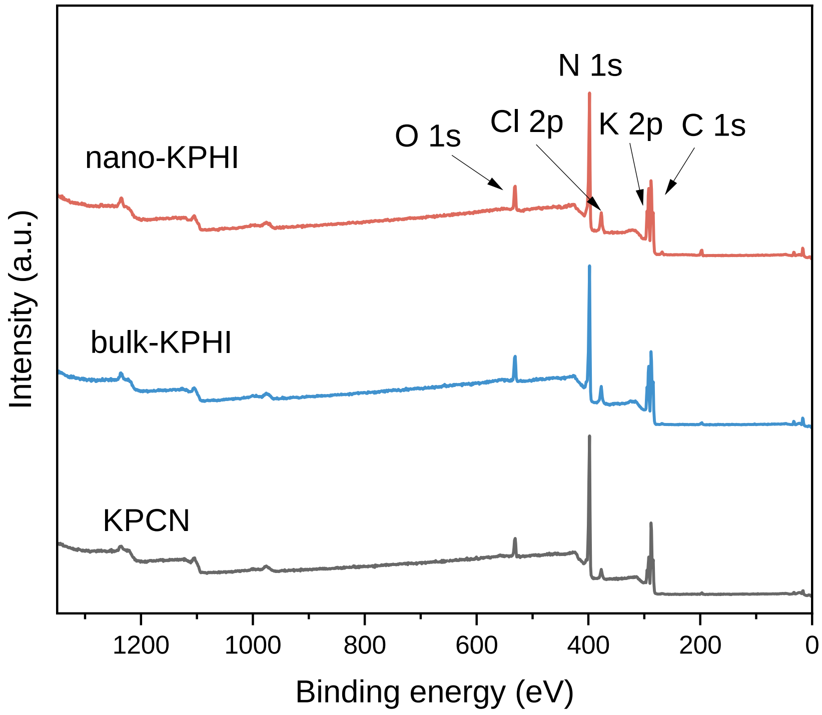

Figure S13. XPS survey of KPCN, bulk-KPHI, and nano-KPHI.

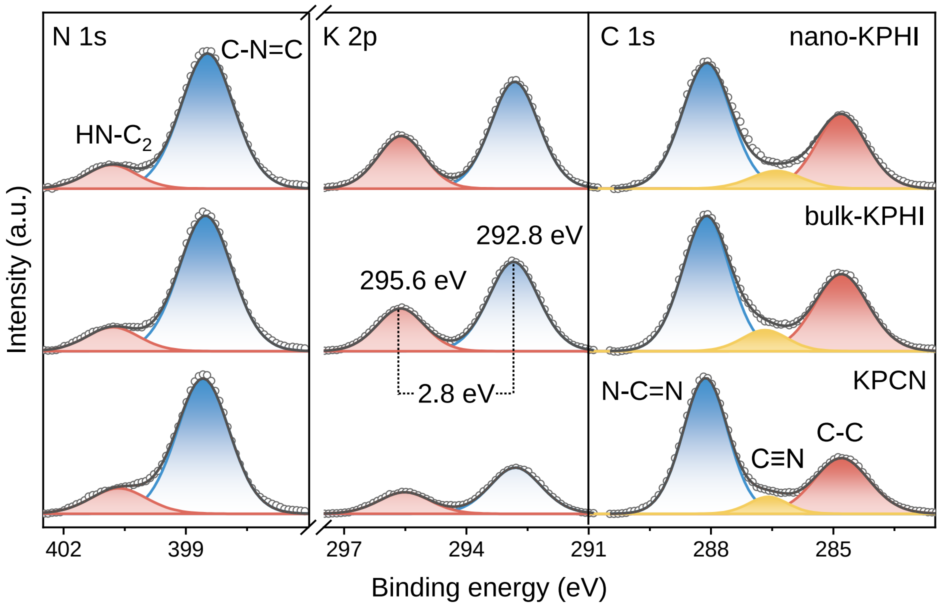

Figure S14. High-resolution XPS spectra for N 1s, C 1s and K 2p of samples.

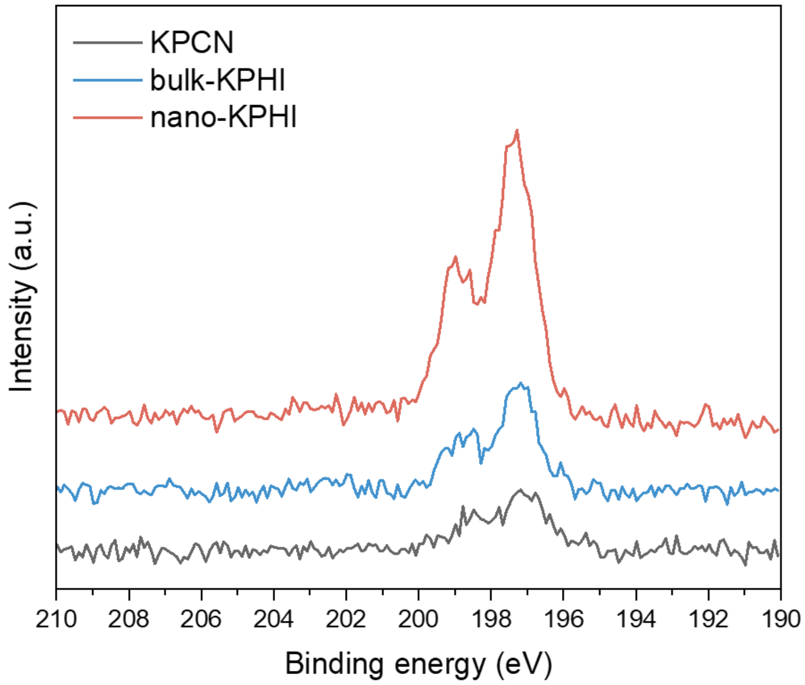

Figure S15. High-resolution XPS spectra for Cl 2p of KPCN, bulk-KPHI, and nano-KPHI.

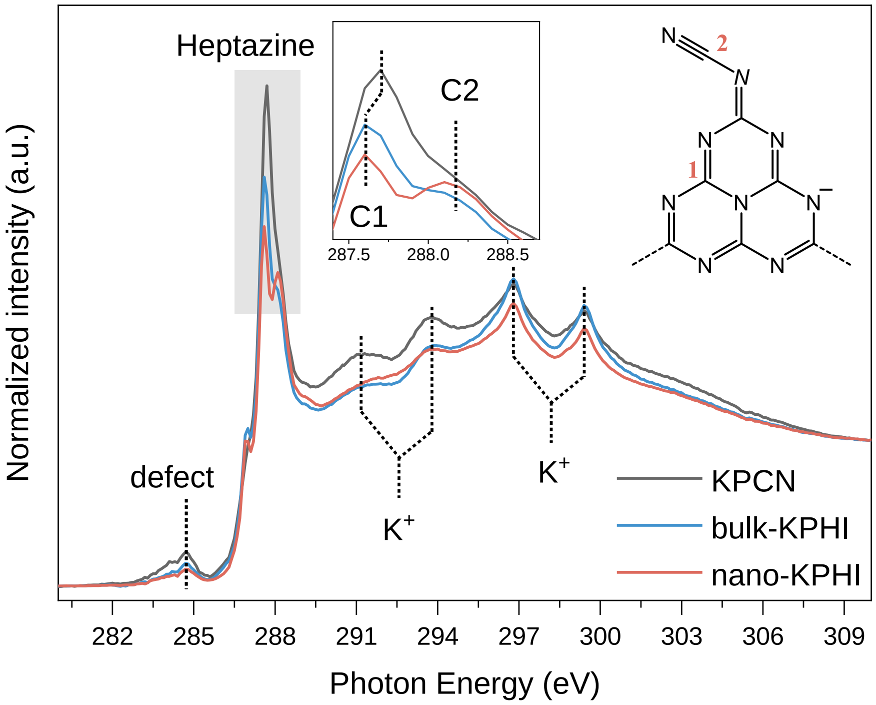

Figure S16. C K-edge X-ray absorption near edge structure (XANES) spectra of KPCN, bulk-KPHI, and nano-KPHI. Inset shows the magnified region of C peaks with labeled carbon atom positions corresponding to different chemical environments in the heptazine units. Peak 1 corresponds to C-N-C coordination (284.9 eV), peak 2 represents C-N=C (285.8 eV), and peak 3 is attributed to N-C=N (287.4 eV). The observed peak splitting and intensity changes reflect differences in electronic structure and local bonding environments among the samples.

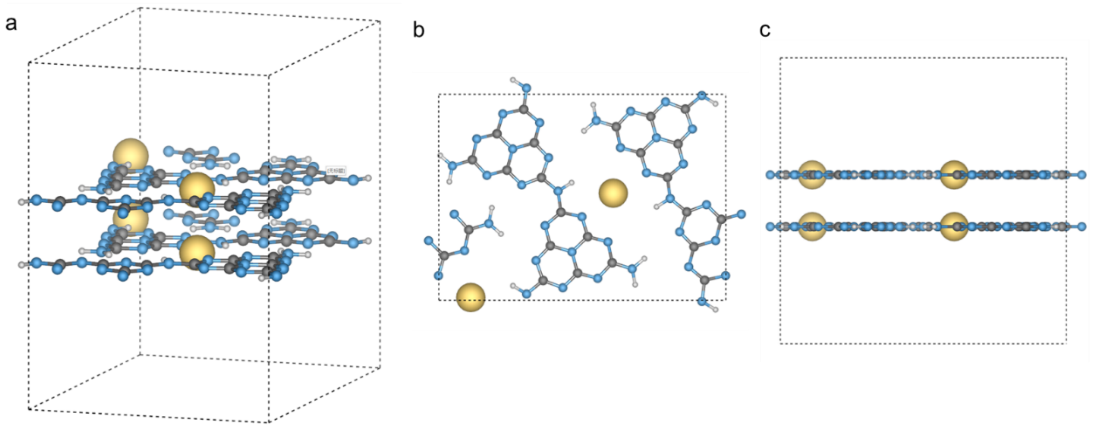

Figure S17. Optimized KPCN crystal cell diagram: **a.** Three-dimensional view, **b.** Top view, and **c.** Side view.

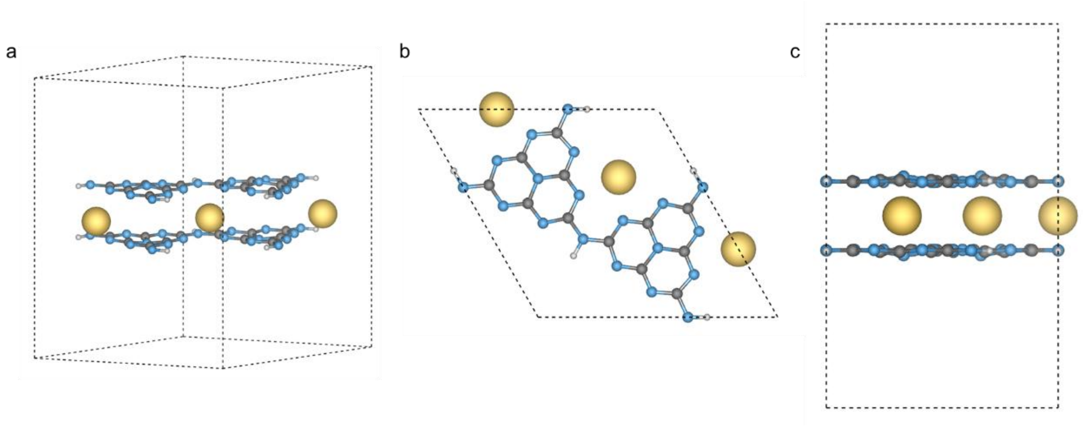

Figure 18. Optimized KPHI crystal cell diagram: a) Three-dimensional view, b) top view, and c) side view.

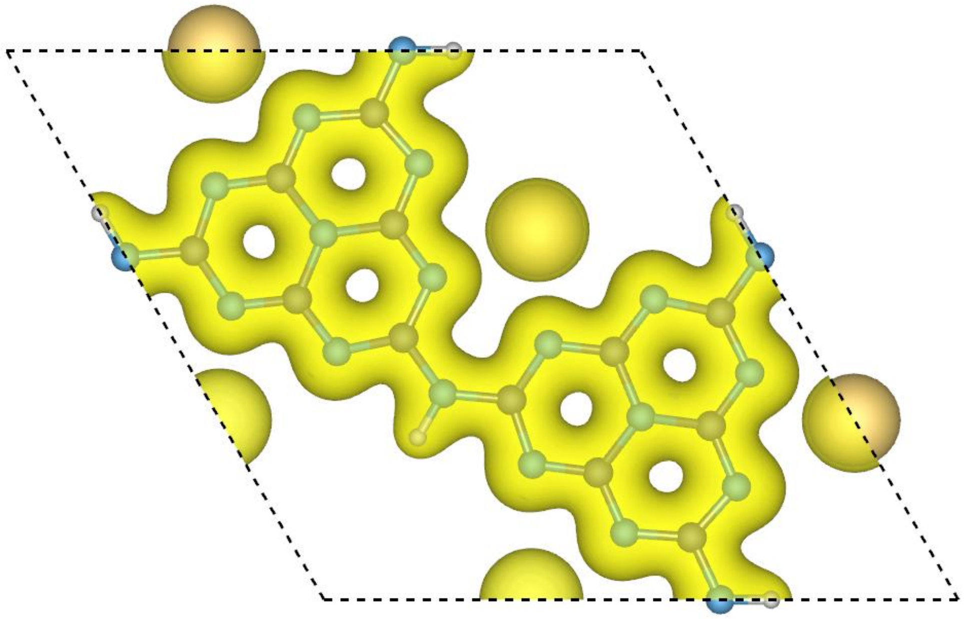

Figure S19. Calculated intralayer charge density of KPHI.

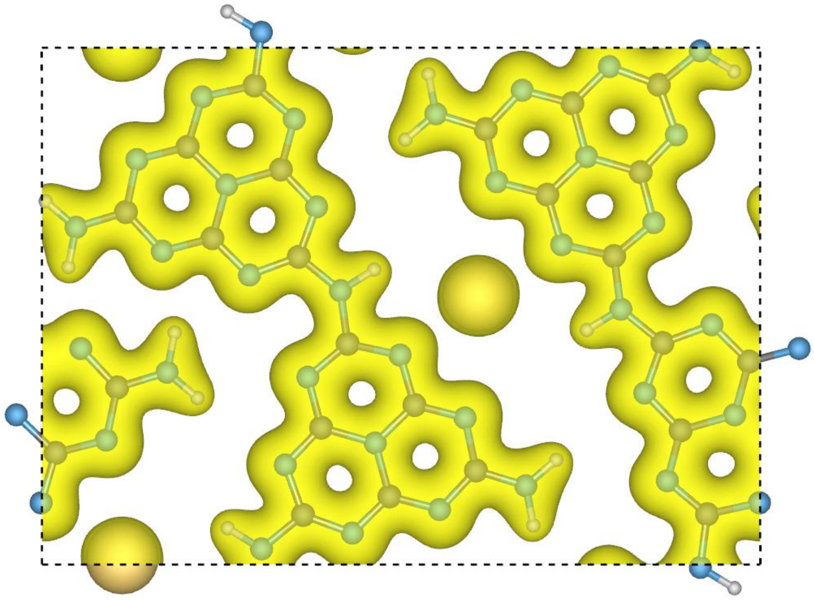

Figure S20. Calculated intralayer charge density of KPCN.

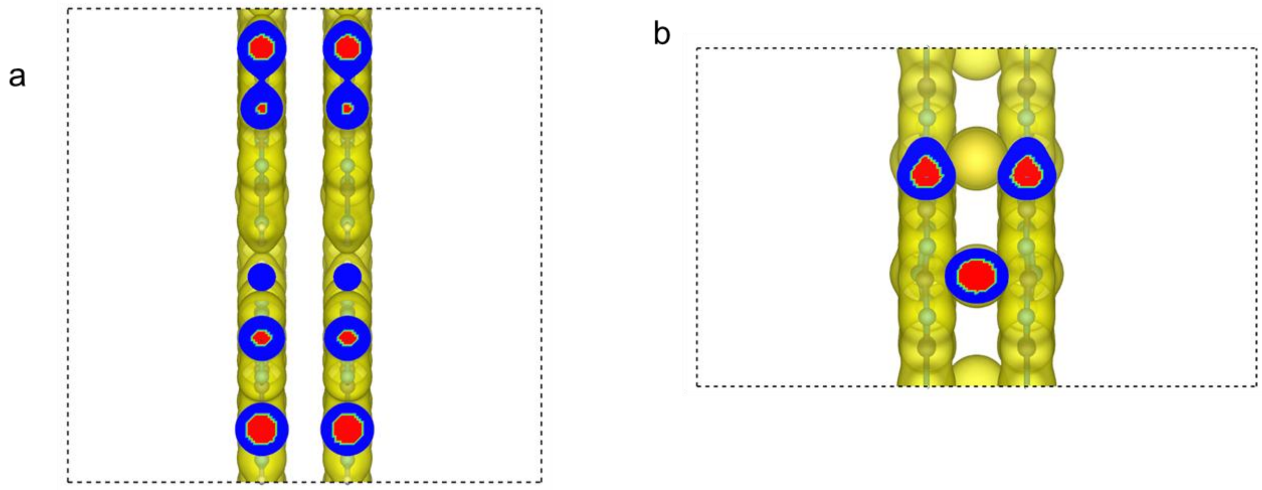

Figure S21. Calculated interlayer charge density of a) KPCN and b) KPHI.

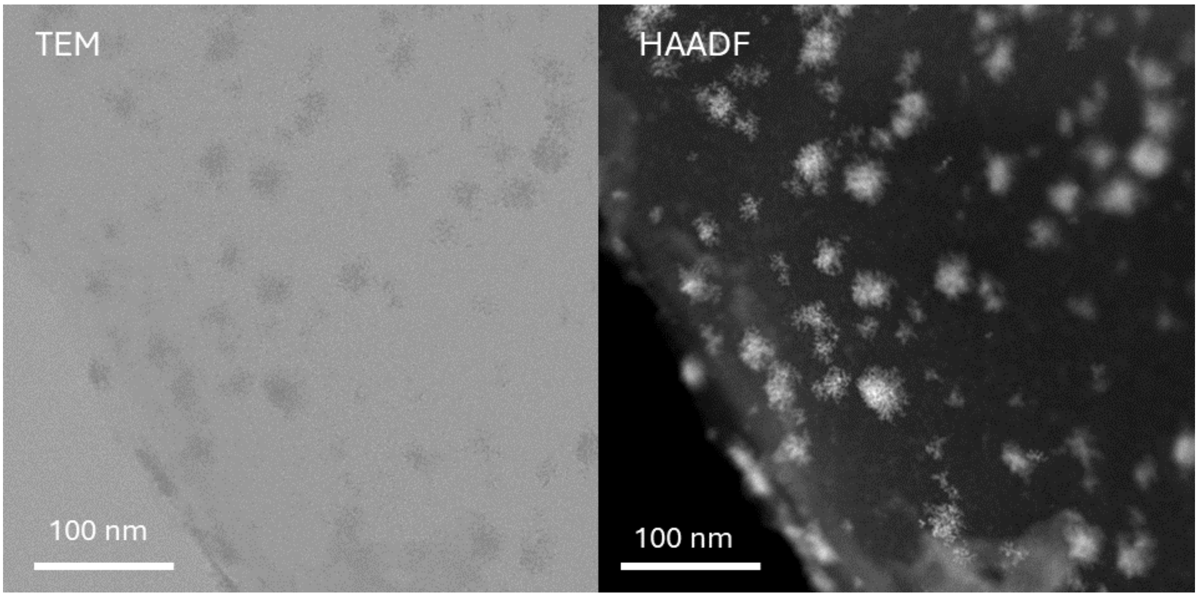

Figure S22. TEM and HAADF-STEM images of KPCN after photodeposition.

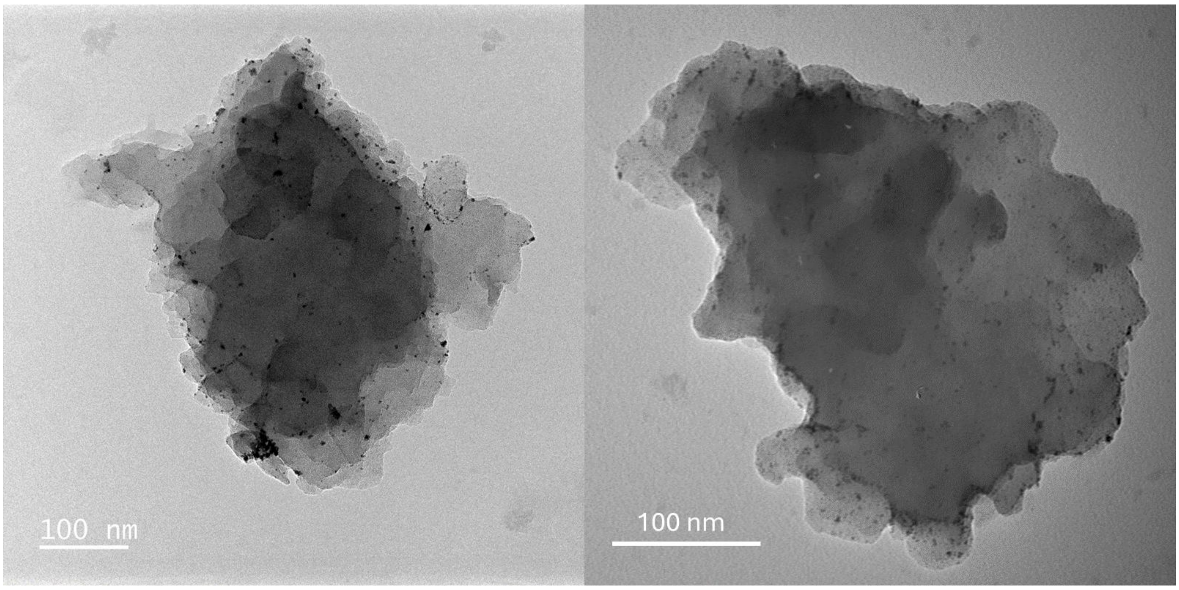

Figure S23. TEM images of bulk-KPHI after photodeposition.

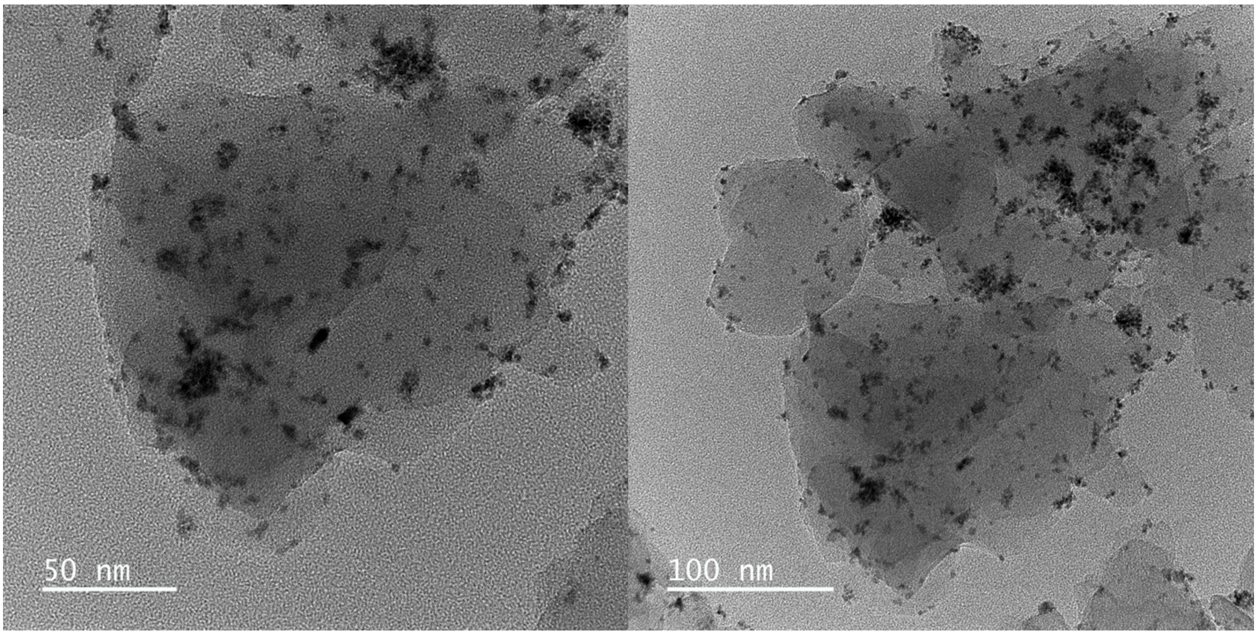

Figure S24. TEM images of nano-KPHI after photodeposition.

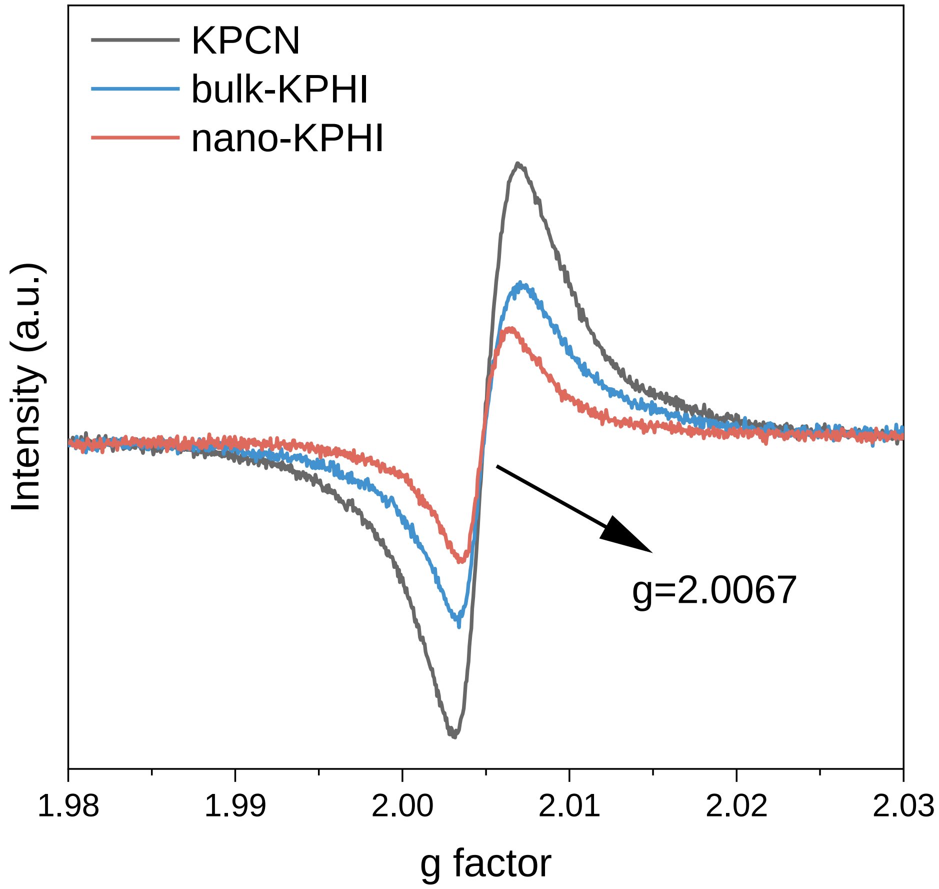

Figure S25. EPR spectra of samples in room temperature.

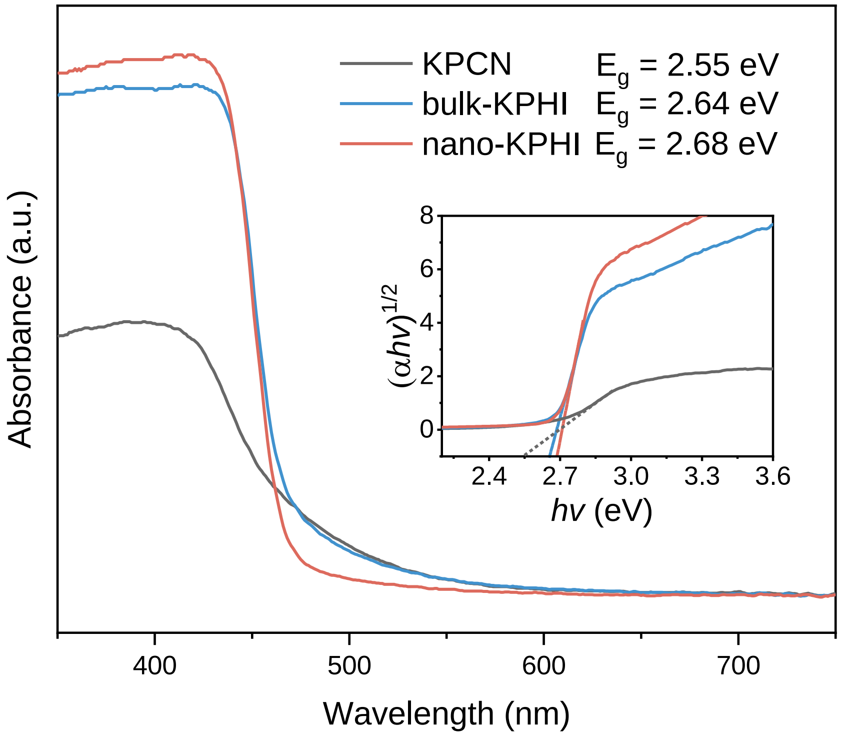

Figure S26. UV-vis absorption spectra of KPCN, bulk-KPHI, and nano-KPHI. Band gap obtained from the UV-vis diffuse reflectance spectra according to the Kubelka-Munk theory (inset).

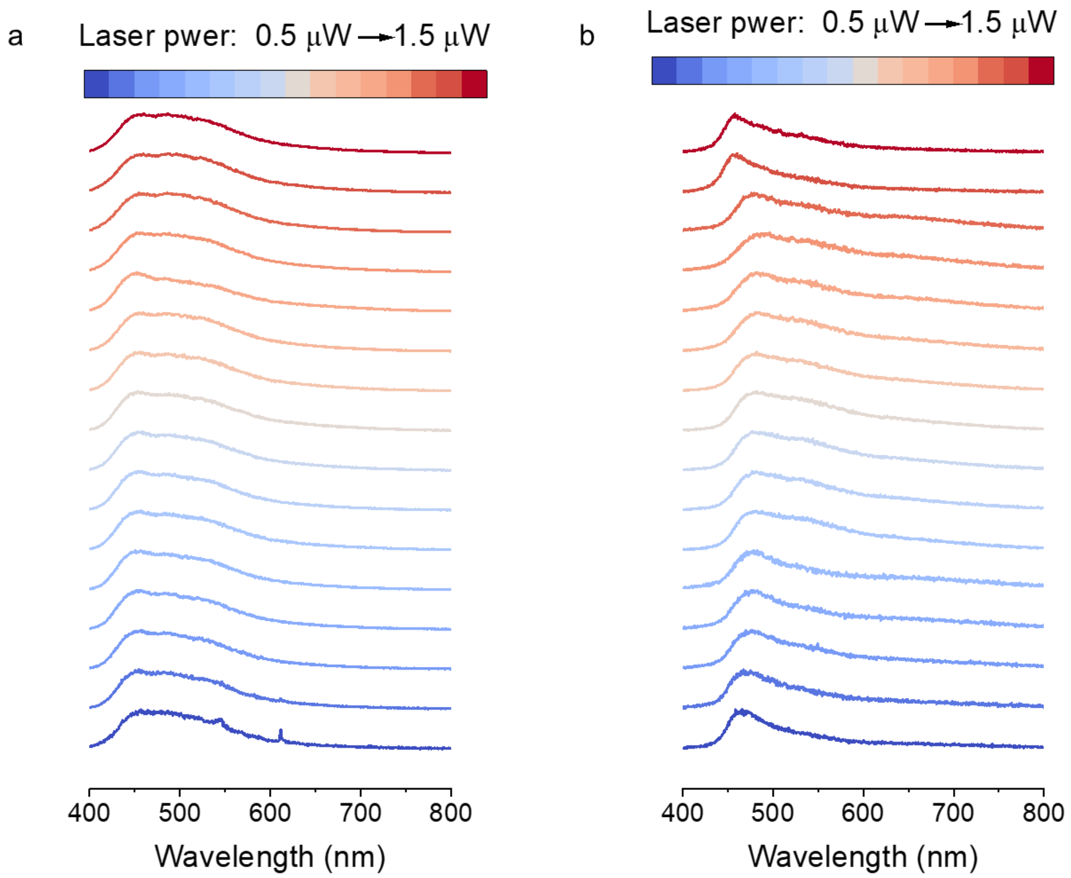

Figure S27. Power-dependent PL spectra of a) KPCN and b) bulk-KPHI showing spectral evolution from 0.5 μW (bottom, blue) to 1.5 μW (top, red).

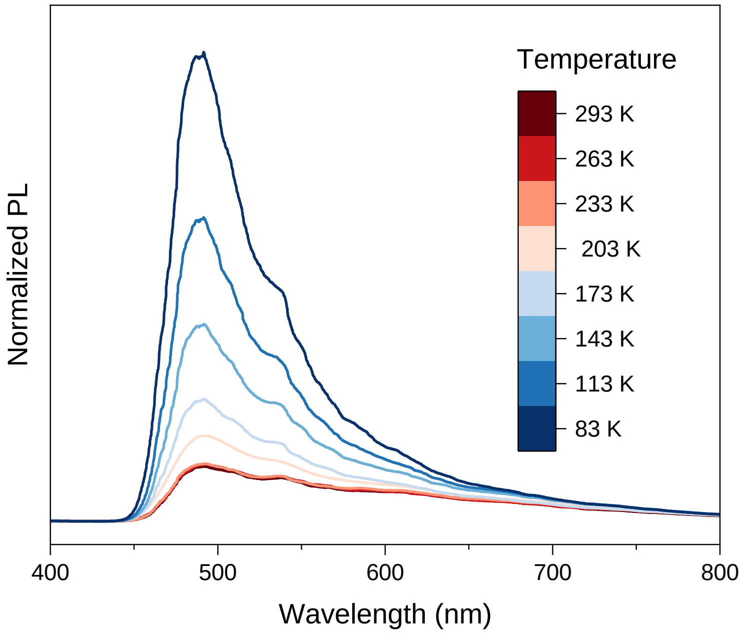

Figure S28. Temperature -dependent PL spectra of nano-KPHI.

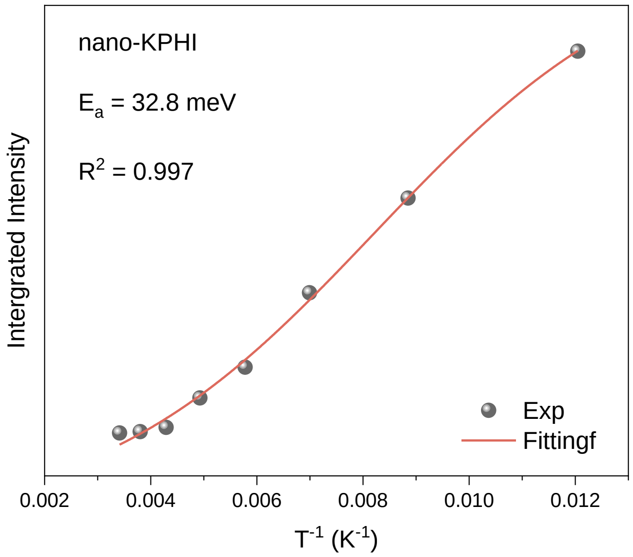

Figure S29. Integrated PL emission intensity nano-KPHI as a function of temperature from 83 to 293 K.

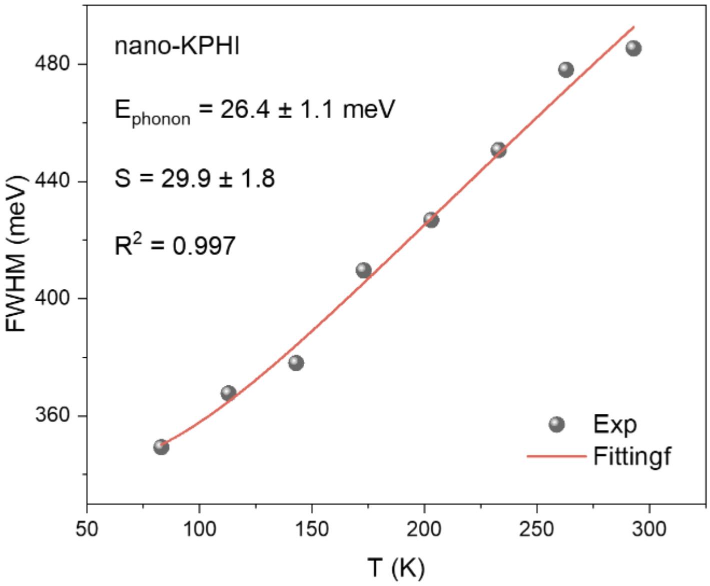

Figure S30. FWHM of STE emission intensity for nano-KPHI as a function of temperature from 83 to 293 K.

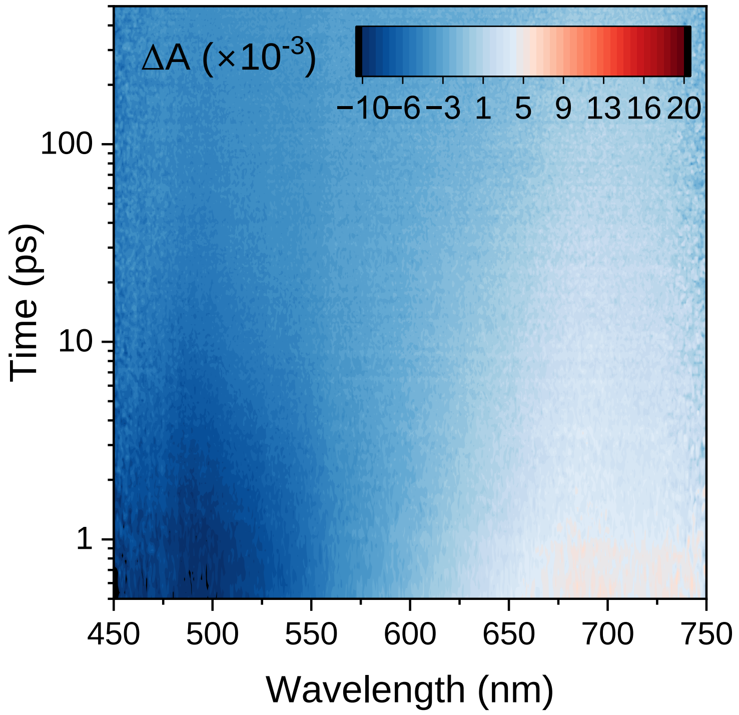

Figure S31. 2D mapping TA spectra of bulk-KPHI under 350 nm femtosecond laser excitation at room temperature in 0-1000 ps.

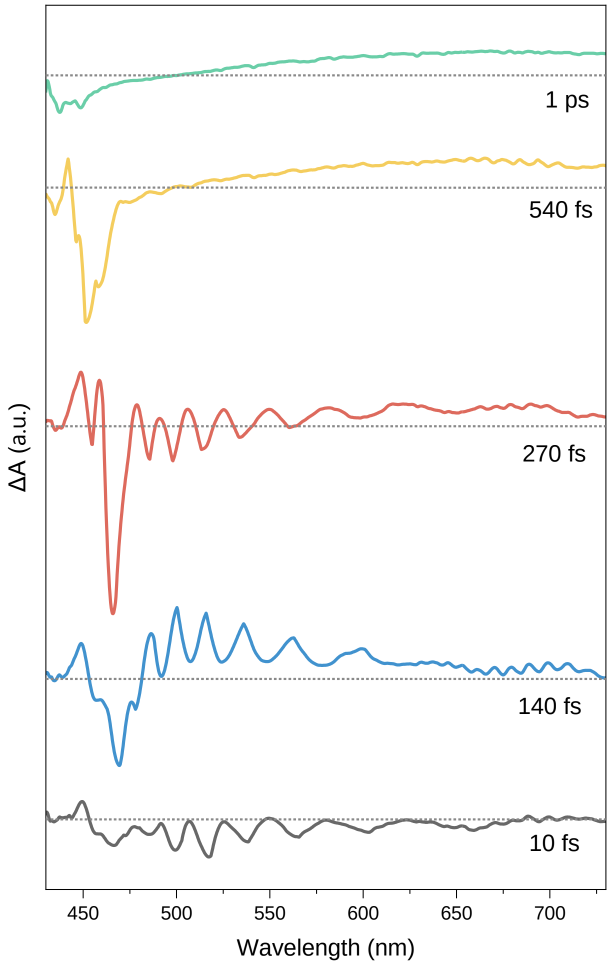

Figure S32. TA spectra of nano-KPHI under 350 nm femtosecond laser excitation at room temperature in 0-1 ps.

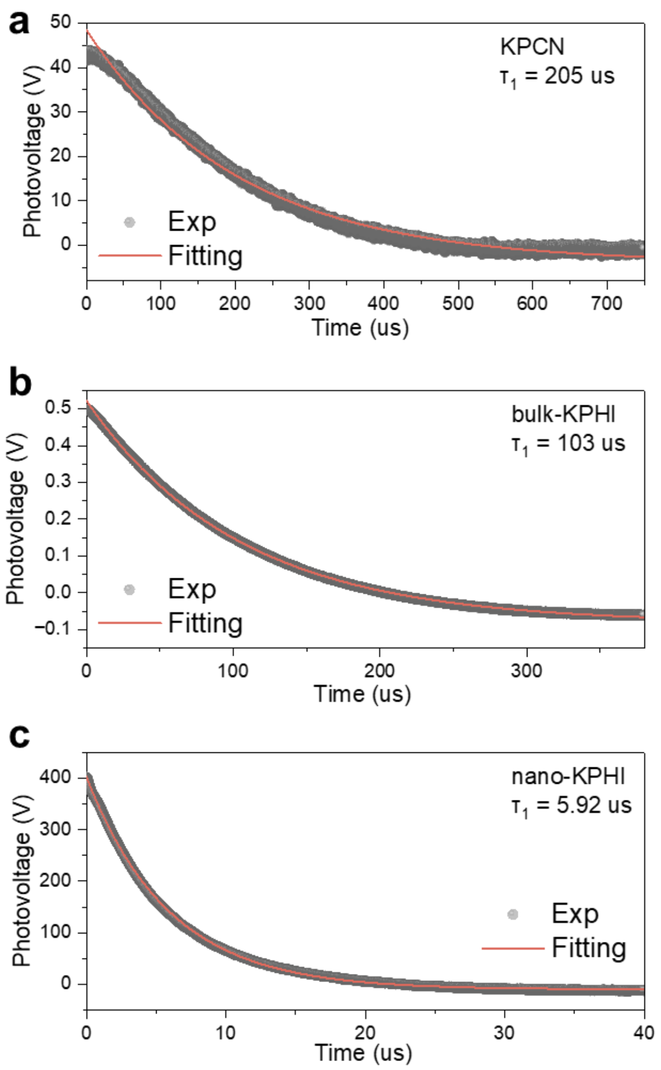

Figure S33. TPV decay curve of a) KPCN, b) bulk-KPHI, and c) nano-KPHI.

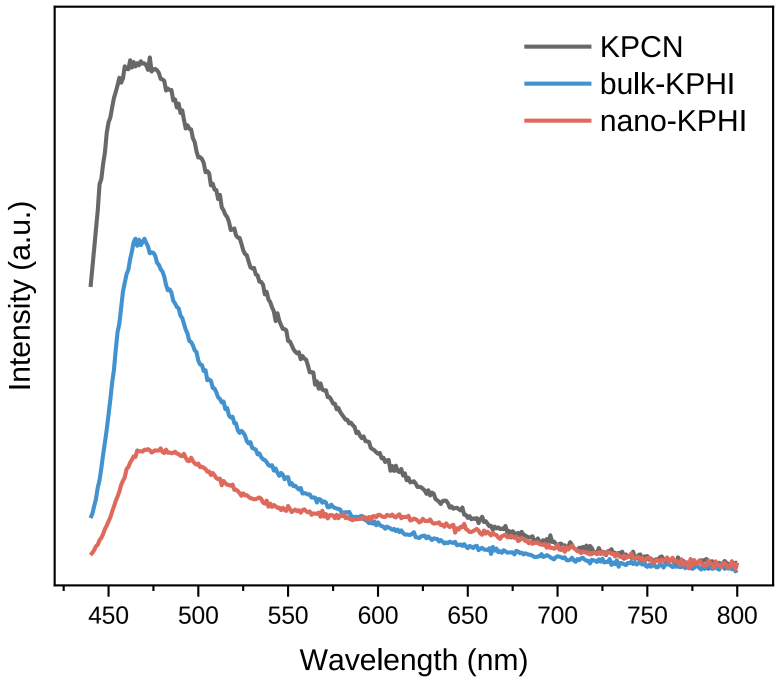

Figure S34. Semi-quantitative comparison of PL spectra of KPCN, bulk-KPHI, and nano-KPHI tested at room temperature.

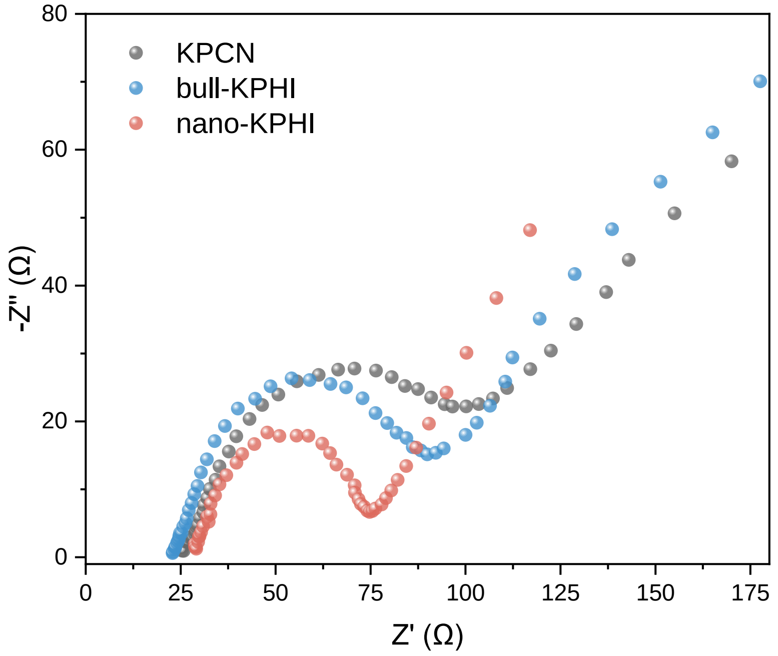

Figure S35. Electrochemical chemical impedance spectra (EIS) of the samples.

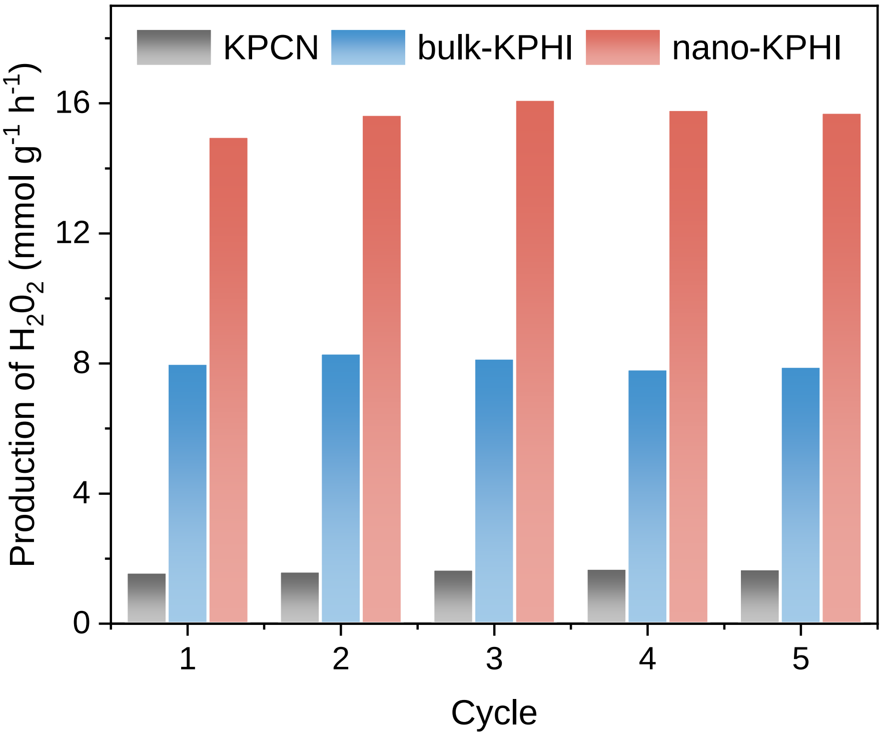

Figure S36. Stability test of Na-PHI of KPCN, bulk-KPHI, and nano-KPHI.

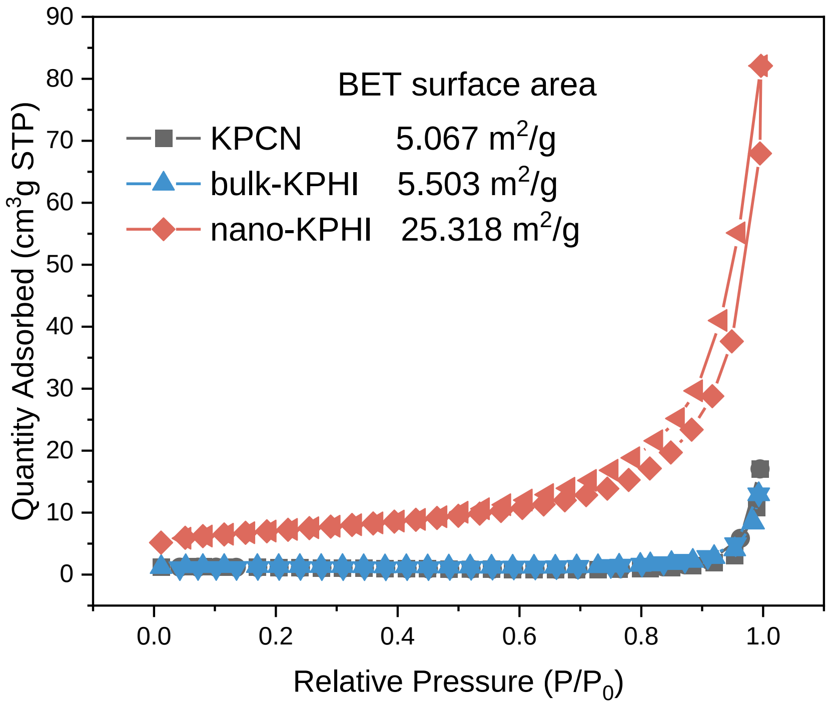

Figure S37. Nitrogen adsorption-desorption isotherm for KPCN, bulk-KPHI, and nano-KPHI.

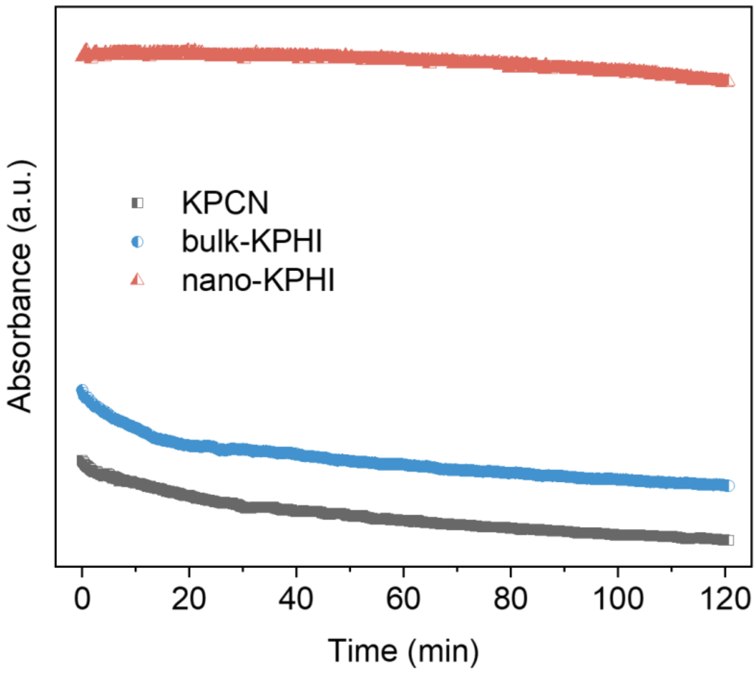

Figure S38. Settling behavior of photocatalysts of KPCN, bulk-KPHI, and nano-KPHI (Actual absorbance changes over time).

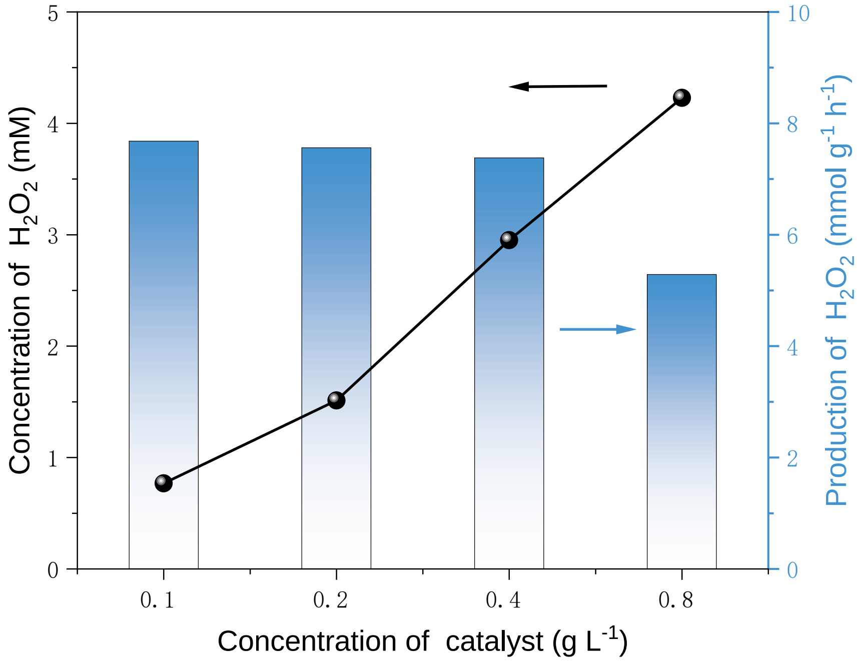

Figure S39. H_2O_2 production rate of bulk-KPHI at varying catalyst loadings.

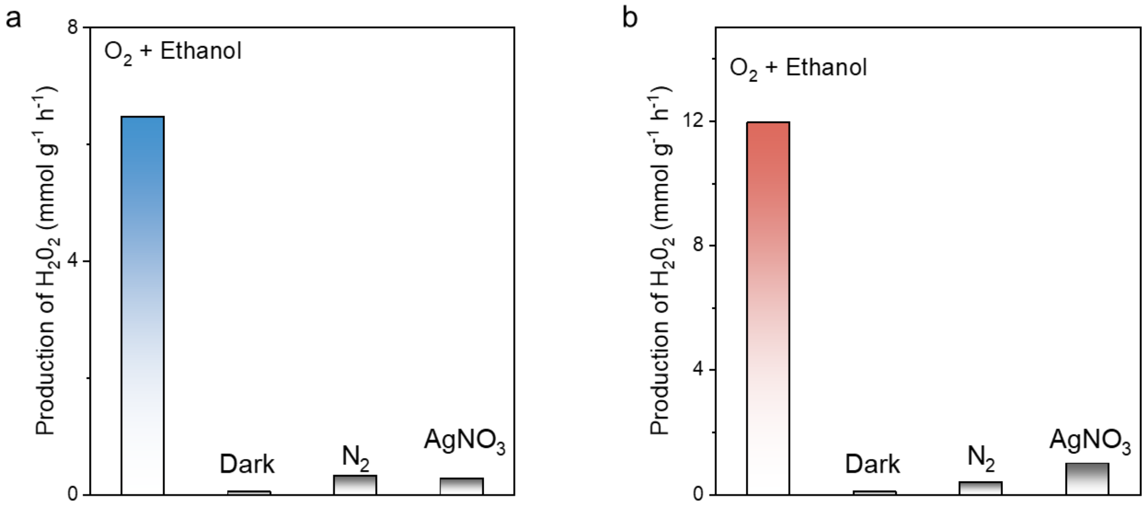

Figure S40. H_2O_2 generation rate of a) bulk-KPHI and b) nano-KPHI under conditions without light or oxygen or with an electron sacrificial agent.

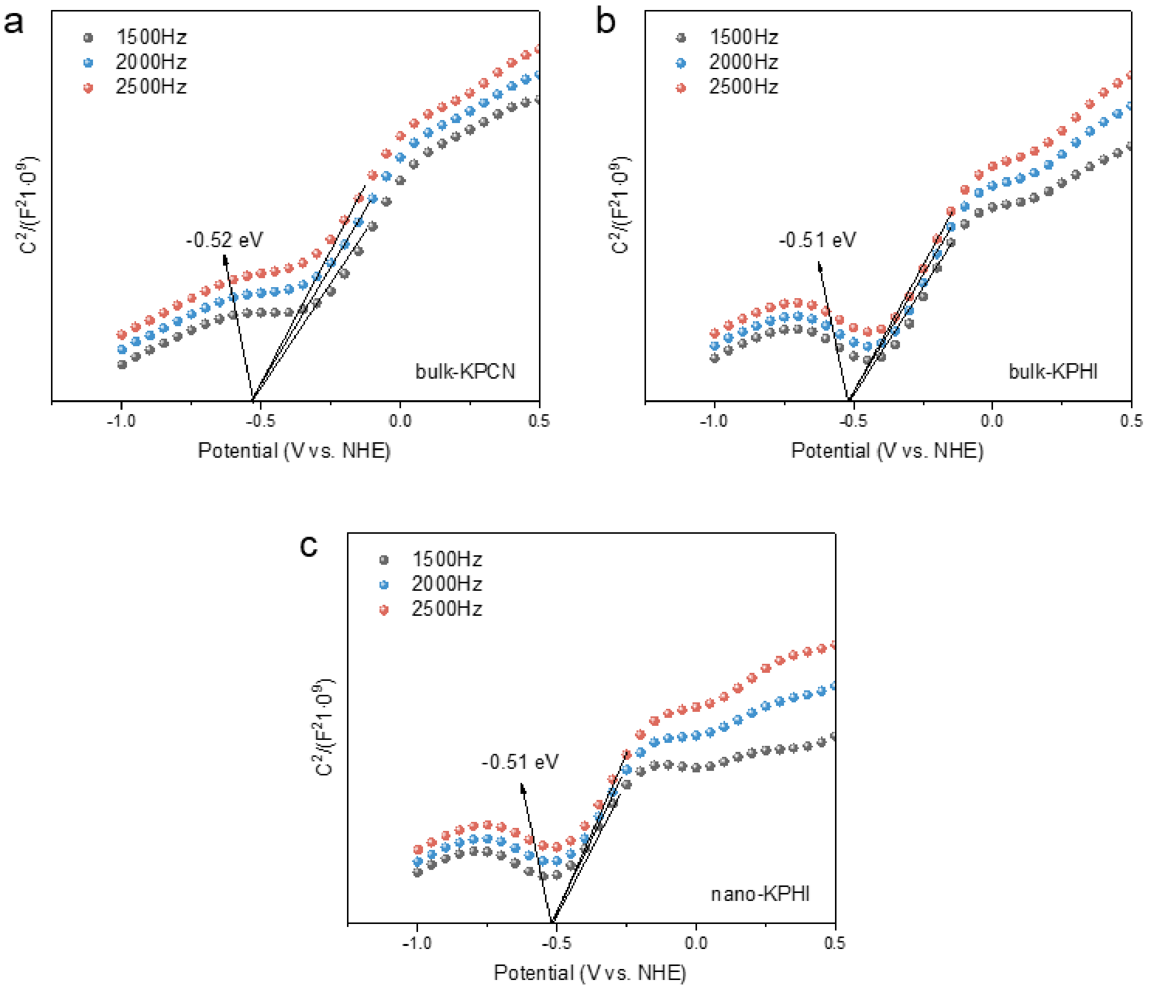

Figure S41. Mott-Schottky curves of **a**) KPCN, **b**) bulk-KPHI, and **c**) nano-KPHI.

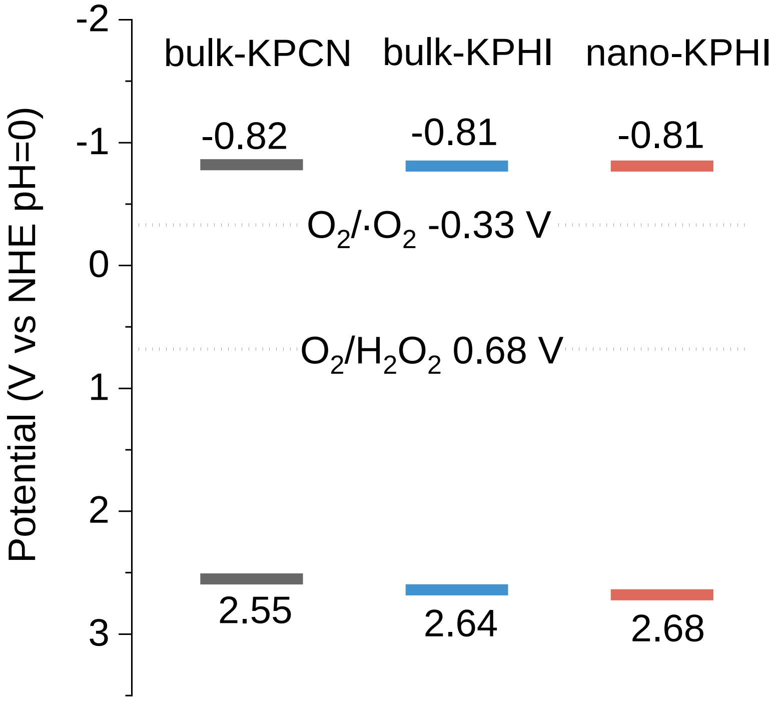

Figure S42. Band structure energy diagrams of KPCN, bulk-KPHI, and nano-KPHI.

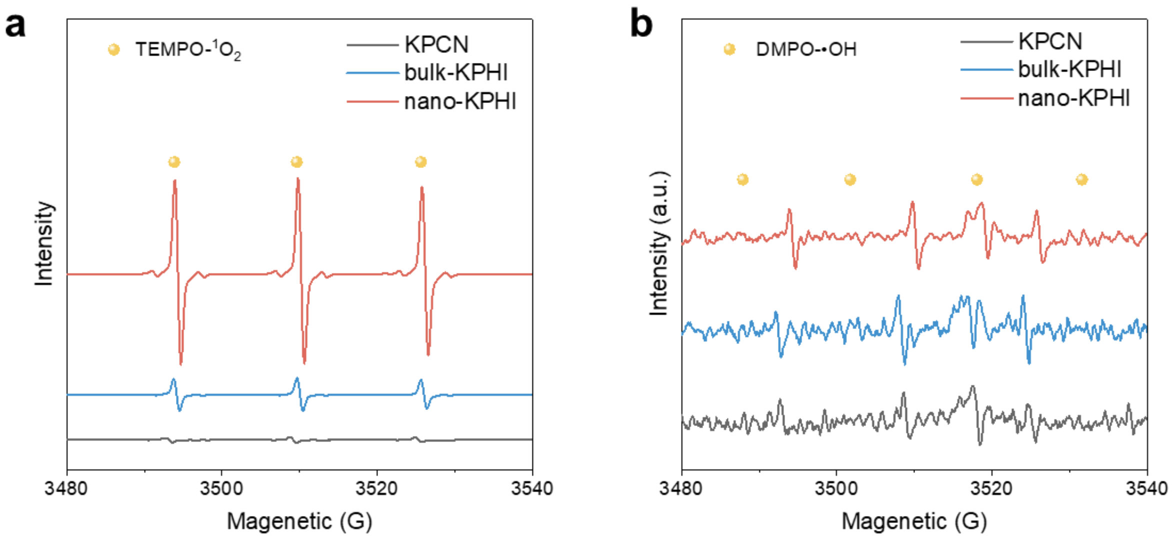

Figure S43. EPR signals of a) TEMPO- $^1O_2$, and b) DMPO- •OH over KPCN, bulk-KPHI, and nano-KPHI.

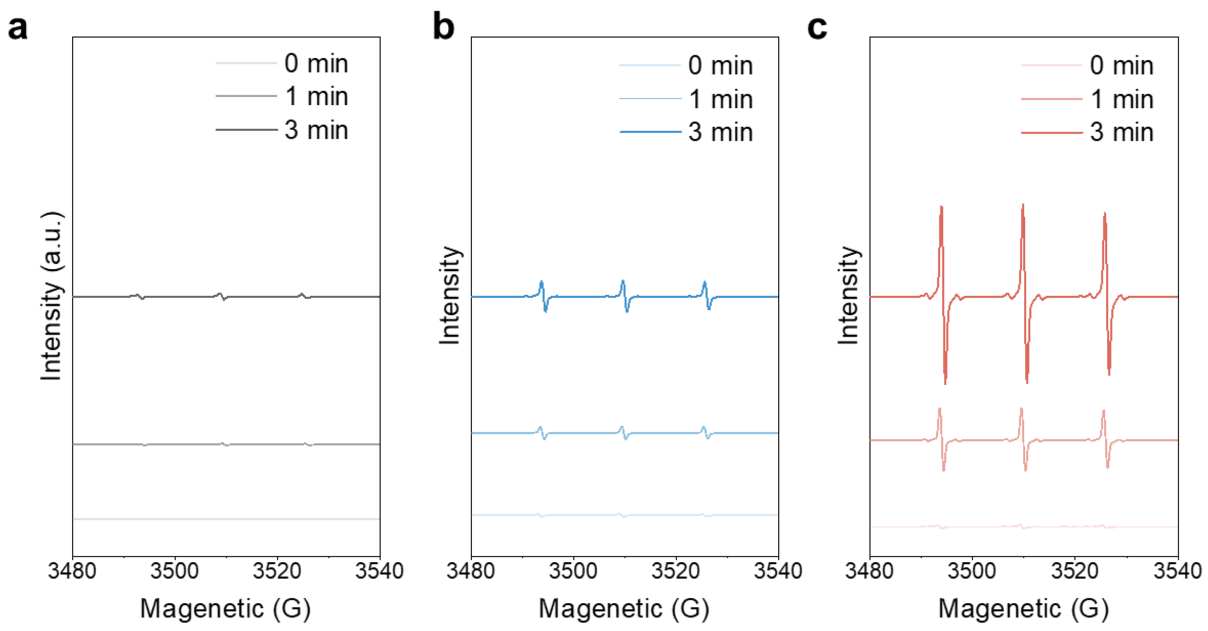

Figure S44. EPR signals of TEMPO- $^1O_2$ over a) KPCN, b) bulk-KPHI, and c) nano-KPHI with different reaction time.

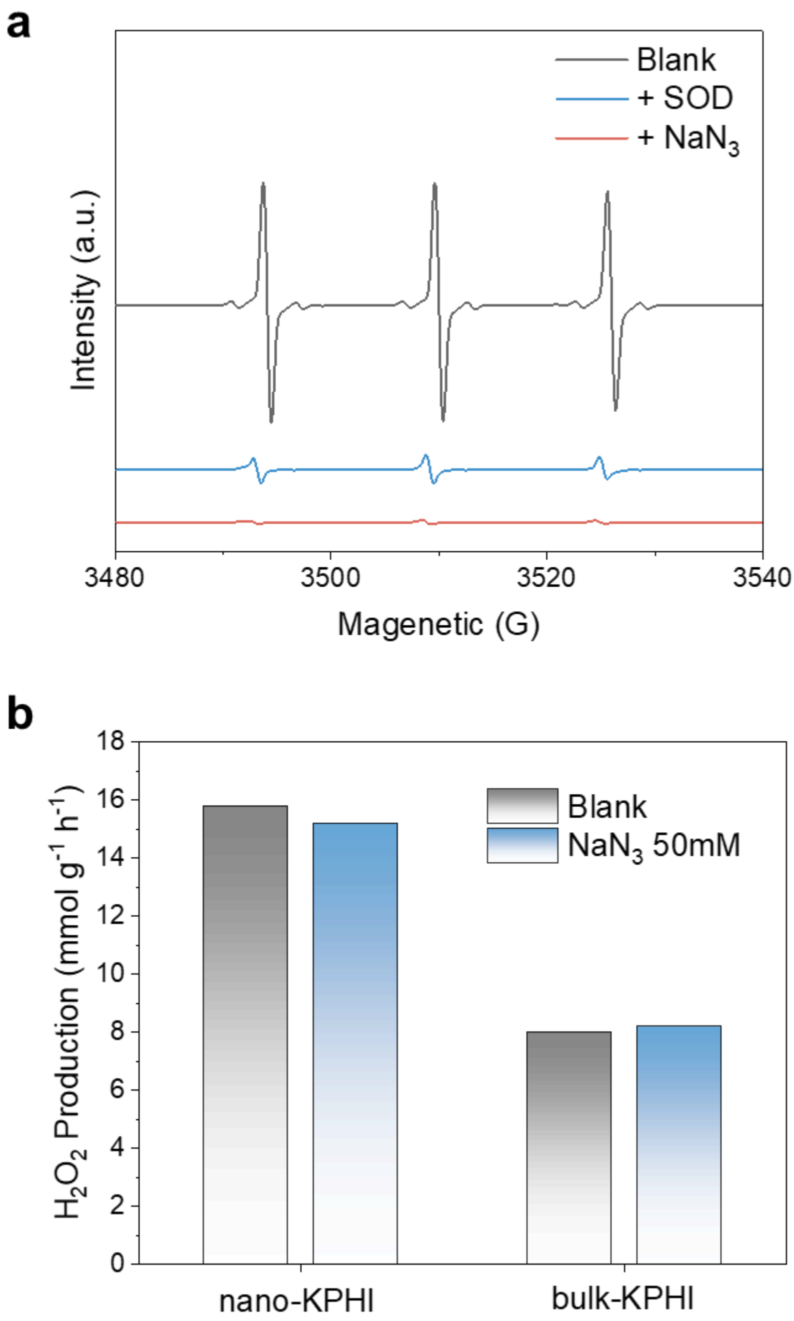

Figure S45. a) EPR signals H_2O_2 generation of nano-KPHI under conditions with SOD or NaN_3. b) H_2O_2 generation rate of bulk-KPHI and nano-KPHI under conditions with NaN_3.

Supporting Tables

Table S1. The fitted decay dynamics of FEs and STEs in nano-KPHI

Component	τ_1 (ps)	A_1	τ_2 (ps)	A_2	τ_3 (ps)	A_3	τ_{ave} (ps)
FE	0.12	-0.22	1.95	-0.03	11.74	-0.01	0.88
STEs	0.40	0.25	3.28	0.14	33.49	0.085	7.02

Table S2. Quantitative analysis results of TPV measurements

Samples	A (a.u.)	Tmax (μs)	τ_1 (μs)	A_{eff}/a.u.
KPCN	1714	46.64	205.06	7566
bulk-KPHI	2036	4.76	103.91	44926
nano-KPHI	187	0.86	5.91	1292

Table S3. Activity comparison between nano-KPHI and other carbon nitride based photocatalysts for H_2O_2 production

Photocatalyst	Irradiation condition	Sacrificial agent	H_2O_2 Production (mmol g^{-1} h^{-1})	Ref.
CN	λ ≥ 420 nm	ethanol	0.125	[6]
Cv-g-C_3N_4	λ ≥ 420 nm	ethanol	0.16	[7]
Porous GCN	λ ≥ 420 nm	ethanol	0.187	[8]
ultra-thin CN	λ ≥ 400 nm	isopropanol	0.665	[9]
Au/CN	λ ≥ 420 nm	ethanol	1	[10]
O-CN	λ ≥ 420 nm	isopropanol	1.2	[11]
CNKPF	λ ≥ 420 nm	ethanol	2	[12]
Na-PHI	λ ≥ 380 nm	ethanol	3.48	[5b]
K-CN	λ ≥ 420 nm	isopropanol	5.50	[13]
Na-C_3N_4	λ ≥ 420 nm	isopropanol	6.53	[14]
C≡N-Na-CN	λ ≥ 420 nm	ethanol	7.01	[15]
ASCN	λ ≥ 420 nm	4-methoxybenzyl alcohol	8	[16]
K/Na-CN	λ ≥ 420 nm	isopropanol	10.20	[17]
N/O-CN	λ ≥ 420 nm	isopropanol	11.14	[18]
CN-KCl/KI	λ ≥ 400 nm	isopropanol	13.69	[19]
O/K-CN	λ ≥ 420 nm	isopropanol	15.47	[20]
crystalline CNK	Simulated solar	ethanol	18.5	[21]
Nv-CN	λ ≥ 420 nm	isopropanol	18.6	[22]
Ni-CN	λ ≥ 420 nm	isopropanol	27.11	[23]
Al-C_3N_4	λ ≥ 420 nm	isopropanol	27.5	[24]
**Nano-KPHI**	**λ ≥ 380 nm**	**ethanol**	**35.04**	**This work**

Reference

[1] a) L. Lin, Z. Lin, J. Zhang, X. Cai, W. Lin, Z. Yu, X. Wang, *Nat. Catal.* **2020**, 3, 649. https://doi.org/10.1038/s41929-020-0476-3; b) Y. Guo, Q. Zhou, J. Nan, W. Shi, F. Cui, Y. Zhu, *Nat. Commun.* **2022**, 13, 2067. https://doi.org/10.1038/s41467-022-29826-z

[2] B. C. C. Cowie, A. Tadich, L. Thomsen, *AIP Conf. Proc.* **2010**, 1234, 307. https://doi.org/10.1063/1.3463197

[3] B. Watts, L. Thomsen, P. C. Dastoor, *J. Electron Spectrosc. Relat. Phenom.* **2006**, 151, 105. https://doi.org/10.1016/j.elspec.2005.11.006

[4] E. Gann, C. R. McNeill, A. Tadich, B. C. C. Cowie, L. Thomsen, *J. Synchrotron Radiat.* **2016**, 23, 374. https://doi.org/10.1107/S1600577515018688

[5] a) P. Zhang, Y. Tong, Y. Liu, J. J. M. Vequizo, H. Sun, C. Yang, A. Yamakata, F. Fan, W. Lin, X. Wang, W. Choi, *Angew. Chem. Int. Ed.* **2020**, 59, 16209. https://doi.org/10.1002/anie.202006747; b) J. Yang, H. Yin, A. Du, M. Tebyetekerwa, C. Bie, Z. Wang, Z. Sun, Z. Zhang, X. Zeng, X. Zhang, *Appl. Catal. B Environ. Energy* **2025**, 361, 124586. https://doi.org/10.1016/j.apcatb.2024.124586

[6] Y. Shiraishi, S. Kanazawa, Y. Sugano, D. Tsukamoto, H. Sakamoto, S. Ichikawa, T. Hirai, *ACS Catal.* **2014**, 4, 774. https://doi.org/10.1021/cs401208c

[7] J. Lei, B. Chen, W. Lv, L. Zhou, L. Wang, Y. Liu, J. Zhang, *ACS Sustain. Chem. Eng.* **2019**, 7, 16467. https://doi.org/10.1021/acssuschemeng.9b03678

[8] Y. Shiraishi, Y. Kofuji, H. Sakamoto, S. Tanaka, S. Ichikawa, T. Hirai, *ACS Catal.* **2015**, 5, 3058. https://doi.org/10.1021/acscatal.5b00408

[9] X. Yang, X. Wang, Y. Liu, B. Liang, J. Hu, H. Huang, *Appl. Surf. Sci.* **2022**, 593, 153470. https://doi.org/10.1016/j.apsusc.2022.153470

[10] G. Zuo, S. Liu, L. Wang, H. Song, P. Zong, W. Hou, B. Li, Z. Guo, X. Meng, Y. Du, T. Wang, V. A. L. Roy, *Catal. Commun.* **2019**, 123, 69. https://doi.org/10.1016/j.catcom.2019.02.011

[11] Z. Wei, M. Liu, Z. Zhang, W. Yao, H. Tan, Y. Zhu, *Energy Environ. Sci.* **2018**, 11, 2581. https://doi.org/10.1039/C8EE01316K

[12] S. Kim, G.-h. Moon, H. Kim, Y. Mun, P. Zhang, J. Lee, W. Choi, *J. Catal.* **2018**, 357, 51. https://doi.org/10.1016/j.jcat.2017.10.002

[13] J. Zhang, C. Yu, J. Lang, Y. Zhou, B. Zhou, Y. H. Hu, M. Long, *Appl. Catal. B Environ.* **2020**, 277, 119225. https://doi.org/10.1016/j.apcatb.2020.119225

[14] H. Che, X. Gao, J. Chen, J. Hou, Y. Ao, P. Wang, *Angew. Chem. Int. Ed.* **2021**, 60, 25546. https://doi.org/10.1002/anie.202111769

[15] L. Chen, C. Chen, Z. Yang, S. Li, C. Chu, B. Chen, *Adv. Funct. Mater.* **2021**, 31, 2105731. https://doi.org/10.1002/adfm.202105731

[16] Q. Li, Y. Jiao, Y. Tang, J. Zhou, B. Wu, B. Jiang, H. Fu, *J. Am. Chem. Soc.* **2023**, 145, 20837. https://doi.org/10.1021/jacs.3c05234

[17] S. Wu, H. Yu, S. Chen, X. Quan, *ACS Catal.* **2020**, 10, 14380.

https://doi.org/10.1021/acscatal.0c03359

[18] H. Zhao, Q. Jin, M. A. Khan, S. Larter, S. Siahrostami, M. G. Kibria, J. Hu, *Chem Catal.* **2022**, 2, 1720. https://doi.org/10.1016/j.checat.2022.04.015

[19] L.-L. Liu, F. Chen, J.-H. Wu, J.-J. Chen, H.-Q. Yu, *Proc. Natl. Acad. Sci. U.S.A.* **2023**, 120, e2215305120. https://doi.org/10.1073/pnas.2215305120

[20] W. Liu, P. Wang, J. Chen, X. Gao, H. Che, B. Liu, Y. Ao, *Adv. Funct. Mater.* **2022**, 32, 2205119. https://doi.org/10.1002/adfm.202205119

[21] Z. Yang, L. Li, S. Zeng, J. Cui, K. Wang, C. Hu, Y. Zhao, *ACS Appl. Mater. Interfaces* **2023**, 15, 8232. https://doi.org/10.1021/acsami.2c22366

[22] L.-L. Liu, F. Chen, J.-H. Wu, M.-K. Ke, C. Cui, J.-J. Chen, H.-Q. Yu, *Appl. Catal. B Environ.* **2022**, 302, 120845. https://doi.org/10.1016/j.apcatb.2021.120845

[23] Y.-Z. Zhang, C. Liang, H.-P. Feng, W. Liu, *Chem. Eng. J.* **2022**, 446, 137379. https://doi.org/10.1016/j.cej.2022.137379

[24] C. Zhuang, W. Li, T. Zhang, J. Li, Y. Zhang, G. Chen, H. Li, Z. Kang, J. Zou, X. Han, *Nano Energy* **2023**, 108, 108225. https://doi.org/10.1016/j.nanoen.2023.108225
